# Microbial micropatches within microbial hotspots

**DOI:** 10.1371/journal.pone.0197224

**Published:** 2018-05-22

**Authors:** Lisa M. Dann, Jody C. McKerral, Renee J. Smith, Shanan S. Tobe, James S. Paterson, Justin R. Seymour, Rod L. Oliver, James G. Mitchell

**Affiliations:** 1 College of Science and Engineering at Flinders University, Adelaide, South Australia, Australia; 2 School of Computer Science, Engineering and Mathematics at Flinders University, Adelaide, South Australia, Australia; 3 Plant Functional Biology and Climate Change Cluster (C3) at University of Technology Sydney, Sydney, New South Wales, Australia; 4 CSIRO Land and Water Waite Research Institute, Adelaide, South Australia, Australia; Universidad Nacional Autonoma de Mexico Facultad de Quimica, MEXICO

## Abstract

The spatial distributions of organism abundance and diversity are often heterogeneous. This includes the sub-centimetre distributions of microbes, which have ‘hotspots’ of high abundance, and ‘coldspots’ of low abundance. Previously we showed that 300 μl abundance hotspots, coldspots and background regions were distinct at all taxonomic levels. Here we build on these results by showing taxonomic micropatches within these 300 μl microscale hotspots, coldspots and background regions at the 1 μl scale. This heterogeneity among 1 μl subsamples was driven by heightened abundance of specific genera. The micropatches were most pronounced within hotspots. Micropatches were dominated by *Pseudomonas*, *Bacteroides*, *Parasporobacterium* and *Lachnospiraceae incertae sedis*, with *Pseudomonas* and *Bacteroides* being responsible for a shift in the most dominant genera in individual hotspot subsamples, representing up to 80.6% and 47.3% average abundance, respectively. The presence of these micropatches implies the ability these groups have to create, establish themselves in, or exploit heterogeneous microenvironments. These genera are often particle-associated, from which we infer that these micropatches are evidence for sub-millimetre aggregates and the aquatic polymer matrix. These findings support the emerging paradigm that the microscale distributions of planktonic microbes are numerically and taxonomically heterogeneous at scales of millimetres and less. We show that microscale microbial hotspots have internal structure within which specific local nutrient exchanges and cellular interactions might occur.

## Introduction

Prokaryotic communities drive biogeochemical cycling processes within aquatic habitats [1]. Despite the large-scale significance of these activities, many key chemical transformation processes mediated by microbes occur within localised microenvironments [2, 3]. However, aquatic microbial distributions and processes are often measured using bulk phase sampling with a “mean field approach”, whereby large, multi-litre, volume samples are collected and microscale features, occurring at the microliter scale, are effectively averaged out [4–7]. This approach misses microscale hotspots and microenvironments where microbial activity and rates of biogeochemical transformation have been predicted to be orders of magnitude higher than the background average [8–12].

Accumulating evidence suggests that aquatic microbes are often distributed heterogeneously at the microscale, with hotspots in abundance sometimes exceeding background abundance by up to 1–2 orders of magnitude [4]. These hotspots may represent microbial aggregation on particulate organic material [1], associations with phytoplankton cells and zooplankton [13, 14], or behavioural responses to microscale patches of dissolved substrates caused by cell lysis, grazing and excretion events [15]. Microscale aggregations of microbes associated with these processes may be driven by localised growth enhancement or chemotaxis into chemical microniches [1, 8, 16–19]. There is indeed growing evidence that bacteria use behaviour to exploit microscopic ephemeral nutrient patches [3, 8]. Variability in the chemotactic capacity and nutrient preferences of microbes as well as variable growth responses within microenvironments may subsequently lead to microscale partitioning of species [3, 20]. The mechanisms for the generation of recently described coldspots [4, 21], which are areas of low microbial abundance, remain uncertain, with the contribution of viral lysis and localised grazing suggested as possibilities. Lysis events will produce intense local concentrations of viruses, which can spread as local epidemics and produce regions of depleted bacterial abundance.

The presence of hotspots and coldspots are the units of microscale patchiness. The formation and maintenance of these hotspots, whether viral or bacterial, is a balance of aggregation and dispersion [22–24]. Dispersion at the microscale in turbulent environments, such as rivers, is driven by Kolmogorov eddies. These are the smallest possible eddies for a given fluid viscosity [25]. Within rivers, where the viscosity is approaching that of pure water, the length of Kolmogorov eddies is approximately 1–10 mm [26, 27]. When heterogeneities and eddies are the same size, mixing is the most efficient homogenising signal. This is relevant for this study as our sampling interval is at the scale where homogeneity should dominate [23].

Although the high shear of Kolmogorov eddies efficiently erase microbial and nutrient signal gradients [28–30], many eddies are much larger, which allows conditions for clustering [30]. This is due to low shear environments being characterised by eddies with long lifetimes. The lifetimes of these Kolmogorov eddies differ depending on turbulence, with lifetimes of approximately 1,000 seconds and lengths greater than 3 cm within freshwater systems [26, 30]. Shear then helps determine where hotspots and coldspots can form, their size and their lifetime [26, 30–32]. Low shear environments that are characterised by eddies with long lifetimes allow microscale nutrient patch formation and consequently chemotactic swarming of bacteria. The lower size limit of these nutrient patches is controlled by the Batchelor scale, which is the smallest scale that nutrient gradients can occur before being dispersed by diffusion [27, 33]. Chemotactic bacteria are able to exploit ephemeral nutrient gradients above this Batchelor scale, which leads to bacterial abundance hotspots [8, 15].

Although hotspots are a ubiquitous feature of microbial distributions their discrimination has remained largely qualitative, being primarily identified as abundance regions that are ‘elevated above’ or ‘exceed’ background variation across one or two sampling points [7, 34, 35]. In addition, coldspots have lacked a definitive characterisation despite being observed in the microscale distributions of microbial communities previously [7, 12, 36]. However, a quantitative method developed by Dann *et al*. [4] discriminated hotspot, coldspot and background values via rank abundance analysis, separating sample values based on their slope and line of best fit. Hotspots were shown to follow a power law best fit and have steep slopes, whereas coldspots and background values followed linear best fits, with coldspots having steeper slopes than background values [4]. Here we expand on this analysis by using a Chow test to verify structural breaks in the rank abundance distribution, and utilising a more detailed assessment of model fits.

Recently we demonstrated that 300 μl coldspots were more taxonomically similar to hotspots than the background, suggesting coldspots might represent dying hotspots or that hotspots are the growth of coldspots [37]. Whether the result is a hotspot or coldspot, the processes that create these variations steepen local taxonomic gradients, which might lead to gradients of metabolic capacity. Once these gradients form they can become self-reinforcing [38–41] through bacterial chemotaxis and uptake, particularly in aquatic environments where bacteria exhibit high speed motility and high performance chemotaxis [42].

Microbial microscale distributions are often restricted to abundance estimates [4, 7, 8, 10–12], with the occurrence of shifts in community composition changes at these small scales less clear. However, taxonomic heterogeneity has been found among confined 1 μl samples of seawater and oil-confined, ancient water droplets [9, 40], where organisms that are rare in the bulk, background phase occur in regions of elevated local abundance. We have recently shown that 300 μl hotspot, coldspot and background regions in freshwater are taxonomically dissimilar. Here we extend upon these findings with the aim of investigating the extent to which further heterogeneity exists *within* the internal structure of hotspots and coldspots by analysing community composition at the 1 μl scale. We hypothesise taxonomic heterogeneity exists between 1 μl hotspot, coldspot and background subsamples. As these microbial hotspots and coldspots are critical microenvironments for cellular interactions and nutrient exchange, understanding their taxonomic composition will aid further understanding in microbial diversity, environmental heterogeneity and ecosystem function.

## Materials and methods

### Sample collection

Samples were collected from the Murray River, South Australia (-35.1, 139.3) on July 2^nd^, 2014. The Murray River is Australia’s largest river and in addition to its ecological importance provides significant levels of agricultural and domestic water supply, and is characterised by highly regulated flows and turbid waters [43, 44]. At the time of sampling the pH was 7.8, water temperature was 13.6°C, total dissolved solids was 445 mg/L and electrical conductivity was 445 μS/cm. Sampling occurred at the sediment-water interface of the river benthos. Sample collection was achieved using a two-dimensional micro-titre plate described previously, which allows for a series of approximately 300 μl samples to be collected simultaneously [4, 21]. This device allows for the collection of 8 microscale vertical profiles, with each comprising of 12 samples, separated by a distance of 0.9 cm, resulting in a matrix of samples spanning distances of 1.4 cm to 11.3 cm from the sediment-water interface. The sampling device was placed vertically against the respective surface and removed in a vertical motion with a 16 cm x 16 cm glass plate used as a cover to minimise mixing and turbulence when collecting samples. A HydroLab DataSonde probe was used to measure environmental parameters. Specific permission to access the sampling site was not required. The field study did not involve endangered or protected species.

For taxonomic identification, 100 μl samples were collected from each microplate well, aliquoted into cryovials and frozen at -80°C until further analyses. For enumeration, 200 μl samples were collected from each microplate well, aliquoted into cryovials containing glutaraldehyde (0.5% final concentration) to fix bacterial cells and viral particles and stored in the dark at 4°C for 15 minutes. Samples were then quick frozen in liquid nitrogen and stored at -80°C prior to flow cytometric enumeration, which was performed within one week of storage to avoid potential sample deterioration [45, 46].

### Flow cytometry

For flow cytometric enumeration, triplicate samples were prepared according to established methods [43, 46, 47]. Briefly, samples were thawed, diluted 1:100 in Tris-EDTA buffer (0.2 μm filtered, pH 8.0, 10 mM Tris, 1 mM EDTA) and stained with SYBR Green I nucleic acid dye (1:20,000 final dilution; Molecular Probes). Virus-like particle (VLP) counts were optimised by incubating samples in the dark at 80°C for 10 minutes [4, 45, 48, 49]. Reference beads of 1 μm diameter (Molecular Probes) were used as an internal concentration, size and fluorescence standard, and were added to samples in a final concentration of 10^5^ beads ml^-1^ [4, 45, 50].

A FACSCanto II cytometer equipped with blue (488 nm, 20 mW, air-cooled), red (633 nm, 17 mW), and violet (405 nm, 30 mW) lasers and phosphate-buffered saline (PBS) solution sheath fluid, was employed to analyse the samples [4]. Samples were run on a low flow rate for 2 minutes. For each sample, green fluorescence (SYBR I), right-angle light scatter (SSC) and forward-angle light scatter (FSC) were recorded. Flow cytometric cytograms and histograms were exported as FCS 3.0 files and analysed via FlowJo (Tree Star, Inc.) to enumerate prokaryotic and VLP populations [44, 51].

### Data analysis

**Fig 1:**
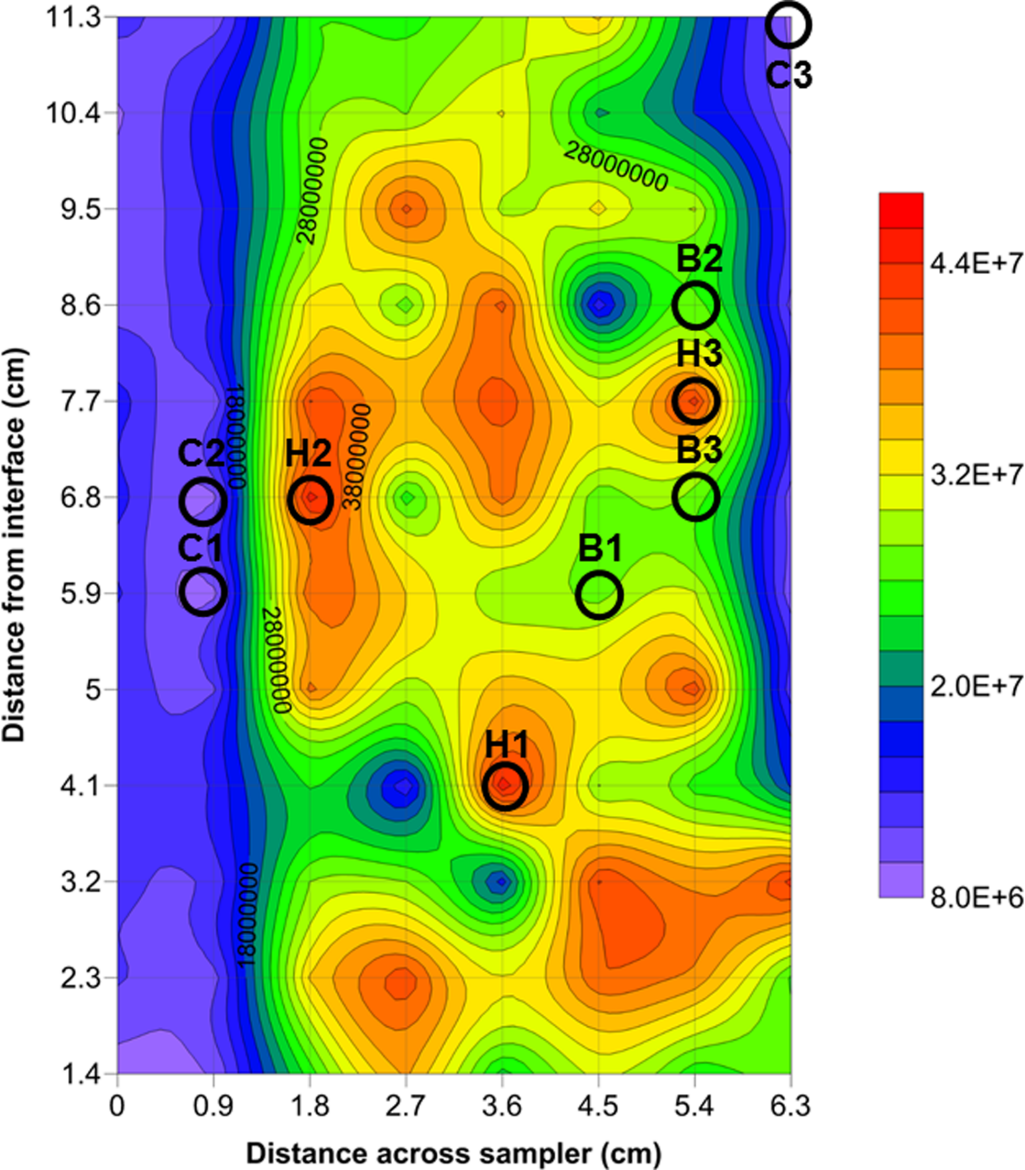
Representative two-dimensional contour plot of total prokaryotic abundance showing hotspots, coldspots and background. Circles indicate sample location and corresponding label. Faint gridlines indicate sampling intervals. Colour intensity scale in cells ml^-1^. Two-dimensional contour plots were created via Surfer 10 (Golden Software, Inc.). Contour plots used a conservative minimum contour interval value of ≥ 1000 events μl^-1^, which was higher than the maximum flow cytometric error of < 24 events μl^-1^.

**Fig 2:**
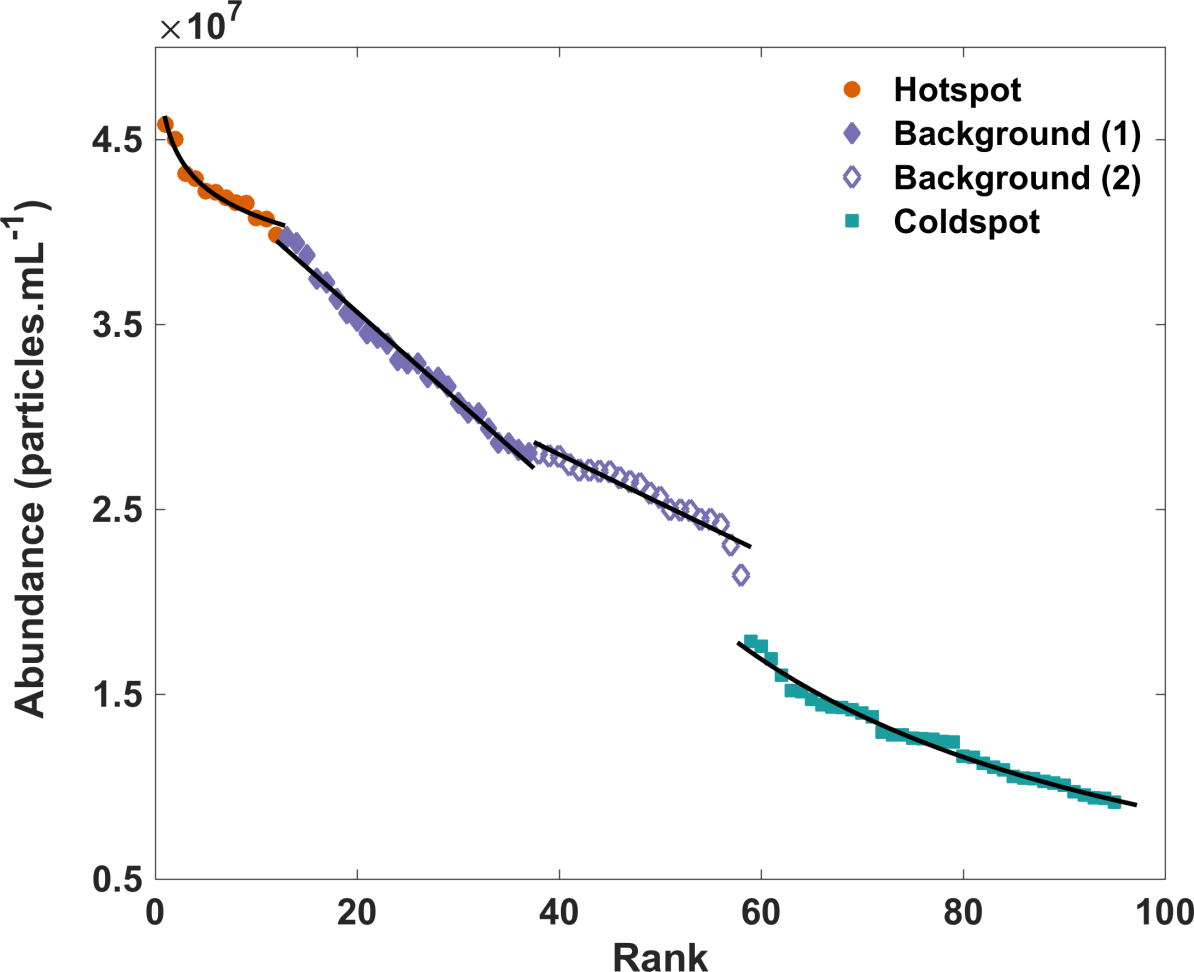
Rank abundance plot showing breaks and fitted distributions for hotspots (power), background (linear) and coldspots (power).

Flow cytometric abundances of prokaryotic and VLP subpopulations were used to construct two-dimensional contour plots in Surfer 10 (Golden Software). A minimum contour interval value ≥1000 events ml^−1^ was chosen when constructing these plots as this was higher than the maximum flow cytometric error, i.e. the background noise within triplicate blank control samples. This value is conservative due to the maximum flow cytometric error being < 24 events ml^-1^. Correlations were performed on these two-dimensional distributions to determine whether there was a relationship between the presence of prokaryotic and viral hotspots and coldspots. Prokaryotic and VLP subpopulation correlations were determined via Pearson’s coefficient with the a of 0.05 reduced via sequential Bonferroni correction (Holm 1979). All possible subpopulation correlations were analysed to determine potential relationships between prokaryotic and viral subpopulations. The spatial relationship of these hotspot, coldspot and background samples are dispersed throughout the sampling area (Figs 1 and S4). Abundance hotspots, coldspots and background regions were defined using rank abundance graphs as described previously [4, 21, 37] with amendments. In order to make distinctions between the regions, two things were necessary: identify the location of breaks in the rank abundance profile, and substantive differences between their distributions, e.g. linear vs nonlinear behaviour. Briefly, flow cytometric total prokaryote abundance counts from the sampling wells were ranked from highest to lowest and assigned ranks. Hotspot regions have previously been identified based on an observed power law distribution [4, 21, 37]. Exploratory regression analysis for the highest 15–20 values on a rank-abundance plot revealed the likelihood of similar behaviour within this dataset. Prior to detailed model fitting, to identify the truncation point between the hotspot and background regions, the top 20% rank-abundance points were log transformed. The optimal breakpoint between the hotspot and background data was found using the *strucchange* package in R (3.7.3), using the ${\text{sup}}_{F}$ criterion for two linear models (maximising the F-statistic, S1 Fig). The same test on non-transformed rank-abundance data was used to identify a split between the background and coldspots, and, as previously observed, an additional split within the background region, creating four distinct sections in the rank abundance profile [4, 21]. All structural breaks were subsequently assessed for significance using a Chow test (R 3.4.3). Regression methods and residuals analysis were then used to fit models to each of the regions (Figs 2 and S2). Given the relatively small spread of the data, and small subsample size introducing the potential for finite-size bias [52], we used MATLAB (R2017b) to compare the fit of three two-parameter models for each region: linear (y = ax+b), exponential ($y\;=\;ce^{\lambda x}$) and power ($y\;=\;cx^{-\beta }$). Fit was assessed by maximising R^2^ values and minimising mean square error (Table 1). Median abundance samples were selected as background rather than the mean, as inclusion or exclusion of the hotspot and/or coldspot values did not overly affect the median values [53]. Using this criteria, three of the highest abundance hotspots (H1, H2 and H3), lowest abundance coldspots (C1, C2 and C3) and median background samples (B1, B2 and B3) were chosen for taxonomic analyses, with five to ten subsamples analysed from each hotspot, coldspot and background sample.

**Table 1:** Selected models for hotspots, background and coldspots together with R^2^ values and square error terms for linear and nonlinear distributions fitted to the same data.

Location & selected model	Model	Sum sq. error	R^2^	Adj R^2^	Mean sq. error
Hotspot: power	Power	1.32E+12	0.9598	0.9558	3.64E+05
$y\;=\;4.61E7∙x^{-0.052}$	Exponential	3.28E+12	0.9007	0.8908	5.72E+05
	Linear	3.49E+12	0.8943	0.8937	5.91E+05
Background (1): linear	Power	4.73E+12	0.9846	0.9839	4.53E+05
y = -4.83E5x+4.53E7	Exponential	2.66E+12	0.9913	0.991	3.40E+05
	Linear	4.34E+12	0.9859	0.9853	4.34E+05
Background (2): linear	Power	7.65E+12	0.8705	0.8637	6.35E+05
y = -2.64E5x+3.85E7	Exponential	6.07E+12	0.8973	0.8919	5.65E+05
	Linear	5.38E+12	0.909	0.9042	5.32E+05
Coldspot: power	Power	3.38E+12	0.9836	0.9831	3.11E+05
$y\;=\;3.59E9∙x^{-1.31}$	Exponential	4.53E+12	0.978	0.9774	3.60E+05
	Linear	7.98E+12	0.9612	0.9601	4.77E+05

### Quantitative PCR

Microplate well samples were analysed via quantitative PCR (qPCR) following methods described previously [37]. Briefly, ten 1 μl water subsamples (R1-R10) were taken for the highest hotspot, lowest coldspot and median background value and five 1 μl subsamples (R1-R5) were taken for the next two highest hotspots, lowest coldspots and median background values. 1 μl water samples were used in each 25 μl PCR reaction consisting of 16S rRNA region-specific forward (27F) and reverse (519R) primers and KAPA Taq Ready Mix 2x (KAPA Biosystems). (519R) were added to 1 μl water samples and stained with Universal KAPA SYBR Fast qPCR Master Mix 2x (KAPA Biosystems). Stained samples were run through 42 cycles on a Rotor-Gene to determine whether sufficient DNA amplification was achieved prior to direct PCR.

### Direct PCR

Direct PCR amplification was performed as described previously [37]. Briefly, ten 1 μl subsamples (R1-R10) were taken for the highest hotspot, lowest coldspot and median background value and five 1 μl subsamples (R1-R5) were taken for the remaining two highest hotspots, lowest coldspots and median background values. 1 μl water samples were used in each 25 μl PCR reaction, which consisted of the 16S rRNA region-specific forward (27F) and reverse (519R) primers and KAPA Taq Ready Mix 2x (KAPA Biosystems). Samples were run for 42 cycles on a Veriti 96 well Thermal Cycler (Applied Biosystems). From each reaction, 20 μl of 16S rRNA amplicon DNA was sequenced whilst the remaining 5 μl was run on an electrophoresis agarose gel to check DNA quality and amplicon size. Negative controls were run for each PCR with sterile filtered water in place of template. PCR products were then run on an agarose gel (2%) to determine relative band intensity and amplification success. Barcoded samples were pooled into equal proportions based on DNA concentrations and molecular weight and were then purified with calibrated Ampure XP beads. Amplicons were then used for 2- by 300-bp Illumina MiSeq sequencing (Molecular Research, Shallowater, TX, USA), which was performed following the manufacturer’s guidelines.

### Bioinformatics

The resulting sequenced bacterial DNA was joined, quality filtered and length truncated with sequences discarded if they contained > 0.5% expected errors for all bases or < 250 bp. Sequences were also discarded if they lacked a recognisable barcode or forward PCR primer. Full length de-replication and abundance sorting using a minimum size of 2 to ensure singletons removal were performed via the USEARCH pipeline [54]. Singleton removal ensured potential spurious OTUs from sequencing artifacts and/or PCR errors were discarded [55, 56]. The cluster_otus command within the UPARSE pipeline in USEARCH v8 was used for OTUs clustering [55]. Reference-based chimeric filtering using the gold database was performed via UCHIME [57, 58]. Reads were then globally mapped to OTUs using a 97% identity threshold. The utax command in USEARCH v8 was used for taxonomic assignment via the RDP Classifier [54, 58, 59]. OTU tables were created via python scripts and the resulting OTU tabbed text file was used to determine average abundances of OTUs. Statistical analyses were performed in PRIMER (Version 7) whereby OTU tables were square root overall transformed and used for similarity percentage (SIMPER) analysis [60, 61]. SIMPER analyses determined the taxonomic groups most responsible for driving dissimilarity within and between samples. Bray-Curtis and Sorensen distance metric [60, 62] resemblance were performed to create dissimilarity matrices for downstream analysis. The Bray-Curtis similarity coefficient between samples 1 and 2 is described by the following equation:

S17=100(1−∑i|yi1−yi2|∑iyi1+∑iyi2)=100.∑imin{yi1,yi2}(∑iyi1+∑iyi2)2

Where $y_{i1}$is the count for the *i*th species from sample 1, and $\sum_{i}\left(\ldots \right)$ is the summation over those species [60, 62].

Sorensen is explained by the following equation:

$$S_{8}=100.\frac{2a}{2a+b+c}$$

Where for a similarity coefficient between samples 1 and 2, *a* represents the number of species present in both samples, *b* represents the number present in sample 1 but absent from sample 2, and *c* represents the numbers absent in sample 1 but present in sample 2 [60, 62]. Bray-Curtis and Sorensen were both used to allow a balance between dominant and rare taxa sensitivity. Specifically, Bray-Curtis is abundance-weighted as it uses species counts, and is therefore primarily driven by the few dominant taxa present, whereas Sorensen weights taxa equally via the use of presence-absence data, therefore being more sensitive to the vast majority of rare taxa present [62]. The dissimilarity matrices generated were then used to perform PERMdisp, which determined the level of dispersion between subsamples [51]. Metric multidimensional scaling (MDS) using bootstrap average analysis was performed to determine the level of spread between samples and produce smoothed 95% bootstrap regions for each sample type. Metric MDS ordination employed 500 bootstrap averages of the centroid of each sample to show where 95% of the centroid averages lay within multivariate space. Presence vs. absence from overall transformed data with Bray-Curtis resemblance was employed to determine subsample exclusivity of bacterial sequences. The OTU abundances were assessed to determine the distribution that best described the taxa abundance profiles for hotspot, background, and coldspot samples. Analysis of the genus abundance distributions for each of the 60 samples was run using OTU raw sequence counts rather than relative abundances to allow for the use of maximum likelihood estimation (MLE). Following current best practise, we (a) only examined fits of models with well-defined probability distributions and (b) used corrected Akaike Information Criterion (AICc) ranks and weights to compare model fits [63]. MLE for each sample was undertaken via the *sads* package in R (3.7.3) for 8 commonly observed ecological abundance distributions. Subsequent fitting of the best performing distribution (Pareto) was done using Clauset et al.’s [52] algorithm, which uses MLE to assess the optimal truncation point $x_{min}\;$and corresponding exponent for the power law distribution $p\left(x\right)\;=\;cx^{-\beta }$ (S1 Table). The association with the Pareto density function, $p\left(X=x\right)=\alpha x_{min}^{\alpha }x^{-\left(1+\alpha \right)}$, where α is the Pareto shape parameter, is given by the simple relationship α = β-1. A goodness-of-fit hypothesis test was then undertaken using the Kolmogorov-Smirnov (KS) test statistic $D_{n}$ against its critical value to assess whether the data fit the model, i.e. accepting the null hypothesis (S2 Table). The KS statistic is given by $D_{n}=\sup_{x}\left|F_{n}(x)-F(x)\right|$, where $F_{n}(x)$ describes the empirical cumulative distribution function and F(x) the cumulative distribution function for the MLE fitted Pareto model. As n≫40, the KS critical values were calculated (sample-wise) by $cv=\frac{\sqrt{-0.5\cdot \text{ln}\left(\frac{\alpha }{2}\right)}}{\sqrt{n}}$, where significance level α = 0.05. Sequences were uploaded to the Harvard Dataverse Network and can be accessed at http://dx.doi.org/10.7910/DVN/SITGAO.

## Results

### Bacterial and viral abundance

Flow cytometric analysis identified 2 prokaryotic (low DNA [LDNA] and high DNA [HDNA]) subpopulations via dense regions in biparametric cytograms of SYBR Green fluorescence and side-scatter (S3 Fig.). Prokaryotic abundances ranged from 5.6 × 10^6^ to 3.6 × 10^7^ cells ml^−1^ (mean = 1.7 × 10^7^, SD = 8.7 × 10^6^, n = 95) for the LDNA subpopulation, from 3.4 × 10^6^ to 1.4 × 10^7^ cells ml^−1^ (mean = 7.4 × 10^6^, SD = 2.8 × 10^6^, n = 95) for the HDNA subpopulation and from 9.2 × 10^6^ to 4.6 × 10^7^ cells ml^−1^ (mean = 2.5 × 10^7^, SD = 1.1 × 10^7^, n = 95) for total prokaryotes. One sample well was empty after water collection, providing a total of 95 samples from the 96-well microplate. Viral abundance was also determined via flow cytometry (See supplementary data).

Rank abundance analysis of the flow cytometric prokaryotic abundances was used to identify hotspots and coldspots (Fig 2, Table 1). Significant structural breaks were present between hotspots and background (p-value 4.1E-9), background and coldspots (p-value <2.2E-16), and within the background region (p-value 3.2E-14). Hotspots and coldspots could also be discriminated from background by the best fitting model being nonlinear–a power law–whereas the background regions were well described by linear models (Figs 2 and S1, Table 1). For background data, there were negligible performance differences between linear and exponential models, with the linear model being slightly better performing in background region 2, and exponential model in background region 1. However, the exponential model parameters were such that the fit was approximately linear within the relevant data range, and in the absence of trends in the residuals, the more parsimonious linear model was chosen (S2 Fig, Table 1). The two-dimensional distribution of prokaryotes was characterised by hotspots and coldspots in abundance (Figs 1 and S4). LDNA had a maximum hotspot of 3.6 × 10^7^ cells ml^−1^ and a minimum coldspot of 3.7 × 10^6^ cells ml^−1^ resulting in a 9.7-fold change in abundance over the sampling area. HDNA had a maximum hotspot of 1.4 × 10^7^ cells ml^−1^ and a minimum coldspot of 2.7 × 10^6^ cells ml^−1^ resulting in a 5.2-fold change in abundance over the sampling area. From one sampling well to the next, the largest change in abundance for LDNA was 5.9-fold, HDNA was 3.7-fold and total prokaryotes was 4.5-fold per 0.9 cm. Hotspots and coldspots were also present in the two-dimensional distribution of viruses (S4 Fig), however, there was no significant correlation between the bacterial and viral subpopulation distributions (p-value ≥ 0.07). The average prokaryotic abundance within the samples used for taxonomic analysis was 2.7 x 10^7^ cells ml^-1^ (SD = 0, n = 3) for the background samples, 4.5 x 10^7^ cells ml^-1^ (SD = 4.5 x 10^7^, n = 3) for the hotspot samples and 0.93 x 10^7^ cells ml^-1^ (SD = 1.2 x 10^5^, n = 3) for the coldspot samples (Fig 1, S3 Table).

### Taxonomic profiles

For the hotspots, a total of 2,127,612 primer matched sequences were quality filtered to yield 1,795,425 (84.4%) passed reads, 4,845 short reads discarded (< 250 bp) and 327,342 low quality records discarded (expected errors > 0.5). Dereplication resulted in 442,770 unique reads and 371,806 singletons. OTU clustering using 97% identity produced 1,601 OTUs and 2,945 chimeras (4.1%). Out of the 1,601 OTUs, reference-based chimera detection identified 47 chimeric reads. Taxonomy assignment via the RDP Classifier produced a total of 1,527 OTUs which contained 1,637,388 total reads. H1 contained between 38,357 and 115,285 reads (mean = 68426, std. dev. = 20971), H2 contained between 46,545 and 114,499 reads (mean = 87493, std, dev, = 26146.7) and H3 contained between 55,989 and 146,072 reads (mean = 103492, std. dev. = 31990.4).

For the coldspots, a total of 1,366,587 primer matched sequences were quality filtered to yield 969,705 (71%) passed reads, 468 short reads discarded (< 250 bp) and 396,414 low quality reads discarded (expected errors > 0.5). Dereplication resulted in 273,700 unique reads and 233,597 singletons. OTU clustering at 97% identity produced 1,322 OTUs and 1121 chimeras (2.8%). Of the 1,322 OTUs, reference-based chimera detection revealed 46 were chimeric reads. Taxonomy assignment via the RDP Classifier produced a total of 1,264 OTUs which contained 879823 total reads. C1 contained between 18,612 and 52,884 reads (mean = 36280, std. dev. = 10679.7), C2 contained between 34,271 and 63,451 reads (mean = 46071, std. dev. = 11920.3) and C3 contained between 9,373 and 121,978 (mean = 57333, std. dev. = 41730.9).

For the background, a total of 1,300,203 primer matched sequences were quality filtered producing 1,007,474 passed reads, 819 short reads discarded (< 250 bp) and 291,910 low quality reads discarded (expected errors > 0.5). Dereplication resulted in 305,310 unique reads and 264,902 singletons. OTU clustering at 97% identity produced 1,299 OTUs and 2,131 chimeras (5.3%). Of these 1,299 OTUs, reference-based chimera detection revealed 46 were chimeric reads. Taxonomy assignment via the RDP Classifier produced a total of 1,244 OTUs which contained 889,419 total reads. B1 contained between 18,344 and 99,216 reads (mean = 68177, std. dev. = 46734.9), B2 contained between 15,399 and 31,150 reads (mean = 23849, std. dev. = 6003.8) and B3 contained between 27,283 and 65,631 reads (mean = 50699, std. dev. = 15027.6).

**Fig 3:**
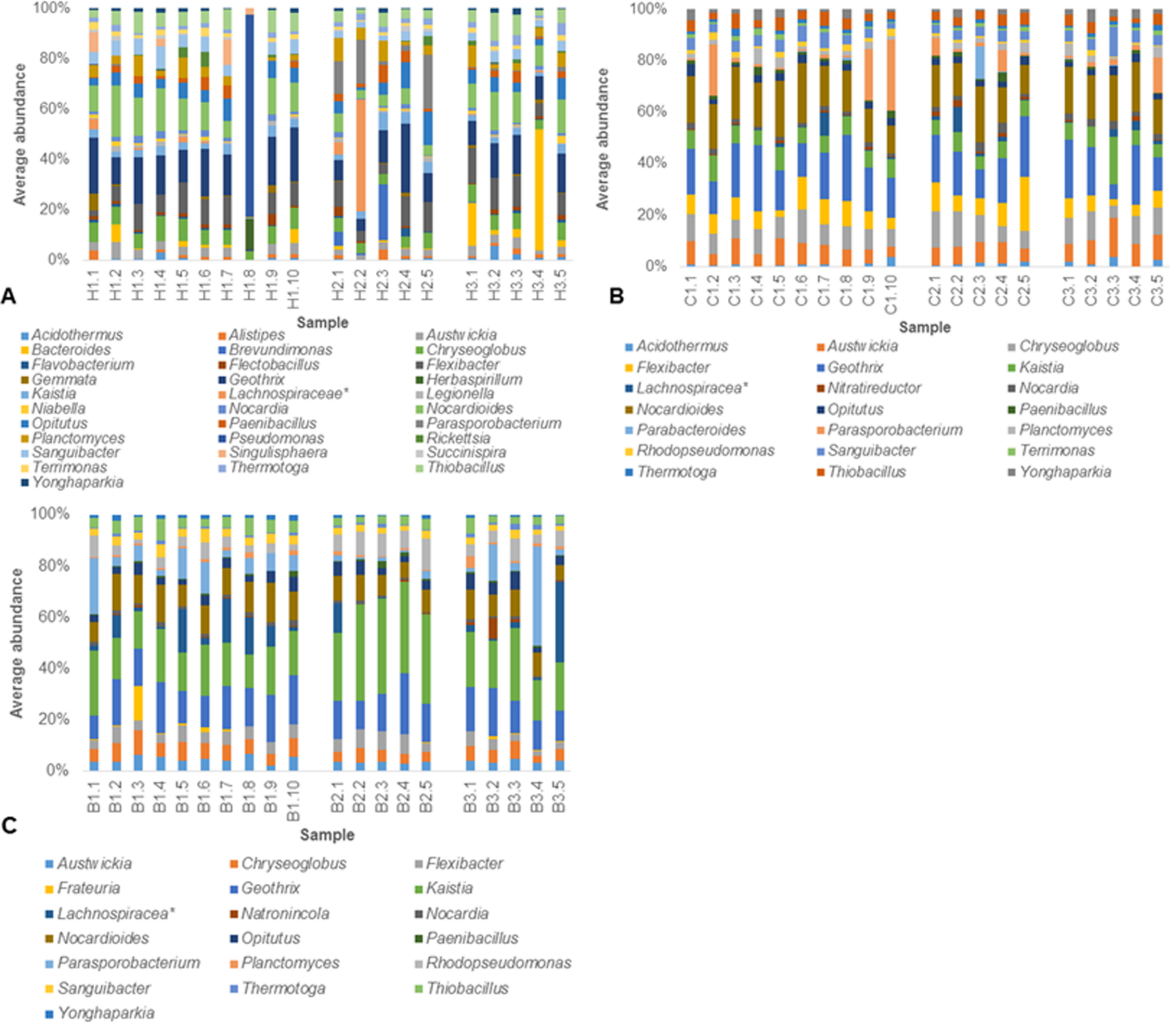
**Genera profiles of (A) hotspot, (B) coldspot and (C) background subsamples.** For clarity, only genera with average abundances ≥ 2% are shown. *incertae sedis. OTUs and associated abundance percentages determined via the RDP Classifier within the UPARSE pipeline [37, 41].

Phylogenetic profiles of the background subsamples showed 95% of subsamples were dominated by Proteobacteria whilst 5% of subsamples were dominated by Firmicutes (S5C Fig). At the genus level, 55% of background subsamples were dominated by *Kaistia*, 25% were dominated by *Geothrix*, 15% were dominated by *Lachnospiraceae* and 5% were dominated by *Parasporobacterium* (Fig 3). At the genus level, SIMPER analysis of the individual samples revealed similarities of 76.2, 74.6 and 74.1 for B1, B2 and B3, with a lower dissimilarity between B1 and B3 (25.5) than B2 and B3 (26.6) and B2 and B1 (27.2) (S4 and S5 Tables). The main drivers for similarity across background subsamples were *Kaistia*, *Geothrix* and *Nocardioides*, which contributed to ≥ 4.2% similarity, whilst the main drivers of dissimilarity among B1, B3, and B2 was *Lachnospiraceae incertae sedis*, which contributed to ≥ 3.7% dissimilarity. PERMdisp analysis at the genus level revealed dispersions of 16.0 (SE 0.2) for B2, 16.4 (SE 0.4) for B3 and 15.9 (SE 0.5) for B1 between the background. No archaea were identified in the taxonomic profiles.

Hotspot subsamples revealed the most dominant phylum was Proteobacteria in 45% of subsamples, Actinobacteria in 35% of subsamples, Bacteroidetes in 15% of subsamples and Firmicutes in 5% of subsamples (S5A Fig). At the genus level, 45% of subsamples were dominated by *Geothrix*, 20% of subsamples were dominated by *Nocardioides*, 10% of subsamples were dominated by *Bacteroides* or *Parasporobacterium* whilst *Brevundimonas*, *Lachnospiraceae incertae sedis* and *Pseudomonas* each dominated a single hotspot subsample (Fig 3). SIMPER analysis of the individual samples revealed similarities of 53.3 for H1, 57.5 for H2 and 61.2 for H3and. Between sample positions there was lower dissimilarity between H2 and H3 (42.9) than H3 and H1 (43.4) and H1 and H2 (46.5) (S4 and S5 Tables). SIMPER analysis revealed the main driver for similarity across the hotspot subsamples was *Geothrix*, which contributed to ≥ 5.5% similarity, whilst the main drivers for dissimilarity were different among hotspots, with *Bacteroides* and *Parasporobacterium* contributing to ≥ 3.2% dissimilarity between H2 and H3, *Parasporobacterium* and *Lachnospiraceae incertae sedis* contributing to ≥ 2.6% dissimilarity between H1 and H2 and *Bacteroides* and *Pseudomonas* contributing to ≥ 2.2% dissimilarity between H3 and H1. PERMdisp analysis at the genus level revealed differences in multivariate dispersion between the hotspots, H1 = 30.7 (SE 5.1), H2 = 26.9 (SE 1.6) and H3 = 24.6 (SE 0.8).

For the coldspot subsamples, phylogenetic profiles showed a dominance of Actinobacteria in 95% of coldspot subsamples and Firmicutes in 5% of subsamples (S5B Fig). At the genus level, 55% of coldspot subsamples were dominated by *Geothrix*, 20% of subsamples were dominated by *Nocardioides* or *Parasporobacterium*, whilst 5% of subsamples were dominated by *Kaistia* (Fig 3). At the genus level, SIMPER analysis of the individual samples revealed similarities of 71.6, 70.3 and 66.0 for C1, C2, and C3, with a lower dissimilarity between C1 and C2 (29.2) than C1 and C3 (31.4) and C2 and C3 (31.7) (S4 and S5 Tables). SIMPER analysis revealed the main drivers for similarity across the coldspot subsamples were *Geothrix* and *Nocardioides*, which contributed to ≥ 4.9% similarity (S4 Table). Comparisons between coldspots revealed the main driver for dissimilarity between C1, C2 and C3 was *Parasporobacterium*, which contributed to ≥ 1.6% dissimilarity. PERMdisp analysis using Bray-Curtis matrices at the genus level revealed significantly different dispersions between coldspots, with a dispersion of 18.8 (SE 0.8) for C2, 19.1 (SE 0.3) for C1 and 21.4 (SE 3.3) for C3.

**Fig 4:**
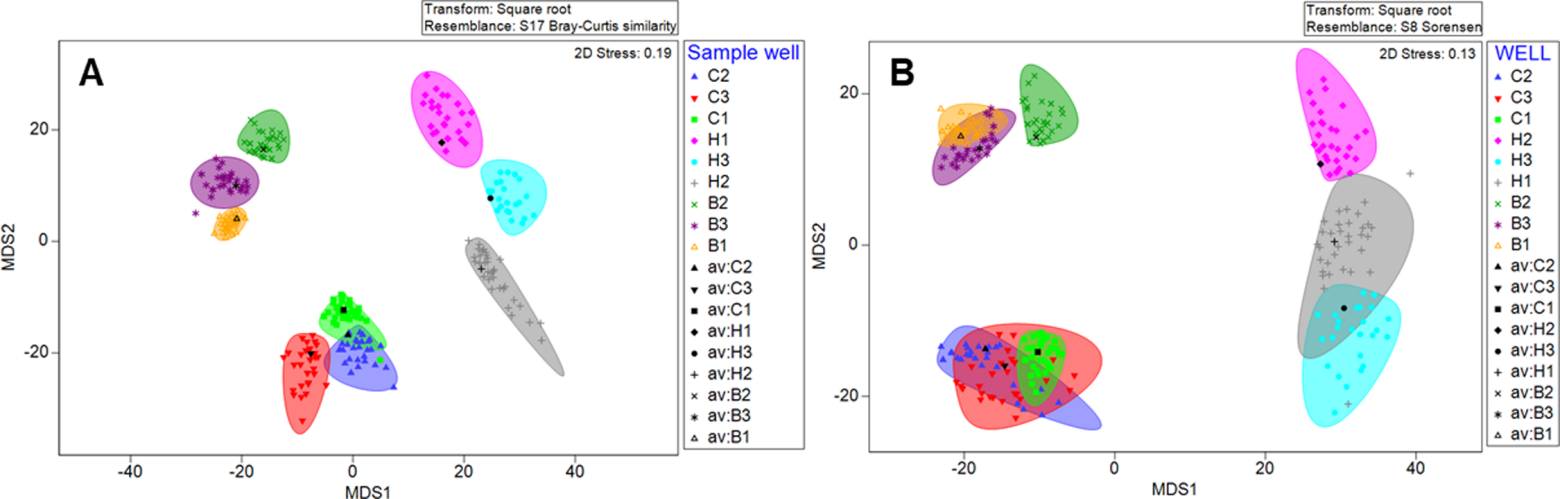
**Metric multidimensional scaling (MDS) analysis using Bray-Curtis dissimilarity (A) and Sorensen distance metric (B) of subsamples from hotspot H1 (pink diamonds), H2 (grey crosses) and H3 (light blue circles), coldspot C1 (green squares), C2 (dark blue triangles) and C3 (red triangles), and background B1 (orange triangles), B2 (green crosses) and B3 (purple stars).** Metric MDS ordination employed 500 bootstrap averages of the centroid of each sample to show where 95% of the centroid averages lie within multivariate space.

Metric MDS analysis using Bray-Curtis matrices showed distinct separation between the background, hotspots and coldspots, with the highest variation observed between hotspot samples rather than coldspot and background samples (Fig 4A). Sample wells grouped together but a higher spread was observed between the hotspot sample wells (Fig 4A). To ensure these groupings were not skewed by the few abundant taxa present, metric MDS analysis was carried out using Sorensen matrices, which although showed an increased overlap between sample region types, yielded similar overall results (Fig 4B).

#### Heterogeneity in taxonomic profiles

Subsample heterogeneity was observed due to heightened abundance of specific taxa, with this heterogeneity most evident in the hotspot subsamples. For instance, Proteobacteria, Firmicutes and Bacteroidetes had heterogeneous increases in relative abundance between hotspot subsamples (S5A Fig). Firmicutes and Proteobacteria also exhibited heightened abundance within the background and coldspot subsamples (S5B–S5C Fig).

Among the Proteobacteria *Pseudomona*s, *Herbaspirillum and Brevundimonas* were the most dominant (Fig 3A). Whereas other hotspot subsamples contained multiple Proteobacteria genera at low abundances, namely *Kaistia* and *Thiobacillus* (Fig 3A). The coldspot subsamples with a heightened relative abundance of Proteobacteria was due to an increase in *Kaistia* (Fig 3B). The heightened abundance of Bacteroidetes within hotspot subsamples was due to *Bacteroides* (Fig 3A).

Among the Firmicutes, *Lachnospiraceae incertae sedis* and *Parasporobacterium* dominated the hotspot subsamples, whilst the remaining subsamples lacking heightened Firmicutes abundance, were dominated by *Paenibacillus* (Fig 3A). The heightened relative abundance of Firmicutes in the background, was due to *Parasporobacterium Natronincola* and *Lachnospiraceae incertae sedis*. *Parasporobacterium* also exhibited heterogeneous dominance in the coldspot subsamples (Fig 3B–3C).

To determine whether the dissimilarity between and within hotspots, coldspots and background was due to the genera exhibiting heightened relative abundance between subsamples, specifically *Pseudomonas*, *Parasporobacterium*, *Lachnospiraceae incertae sedis* and *Bacteroides*, these genera were removed and SIMPER analysis was performed. This resulted in minimal change in average similarities within and between samples suggesting these genera are not responsible for all the dissimilarity between and within sample regions (S4 and S5 Tables). To determine whether the higher heterogeneity observed within the hotspots was due to the specific outlier subsamples from each hotspot, namely H1.8, H2.2 and H3.4, these samples were removed, resulting in higher average similarities within H1 (63.8), H2 (60.4) and H3 (62.9). In addition, comparisons between the hotspots showed a decreased average dissimilarity of 41.1 between H2 and H3, 38.1 between H1 and H3 and 40.2 between H2 and H1 (S4 and S5 Tables). However, after removal of the outlier hotspot subsamples, the level of dissimilarity between hotspots was still higher than that observed in the other sample regions, indicating the outlier subsamples were not the only contributor to the heterogeneity between hotspots and coldspots and background regions.

#### Homogeneity in taxonomic profiles

Acidobacteria, Actinobacteria, Chloroflexi, Nitrospira, Planctomycetes, Thermotogae and Verrucomicrobia were present in all subsamples and had relatively consistent average relative abundances between subsamples (S5 Fig). In addition, Gemmatimonadetes was present in the hotspots and coldspots and Fusobacteria was present in the hotspots (S5 Fig). Of these phyla, SIMPER analysis revealed the genera *Geothrix*, *Nocardioides*, *Flexibacter*, *Austwickia* and *Chryseoglobus*, had abundances that were common amongst subsamples, with the former most dominant for hotspots and coldspots, as well as *Kaistia*, which dominated for background. These genera were the main drivers for similarity between subsamples, hence indicating a common taxonomic community amongst subsamples from hotspot, coldspot and background regions.

### Rank abundance analyses

**Fig 5:**
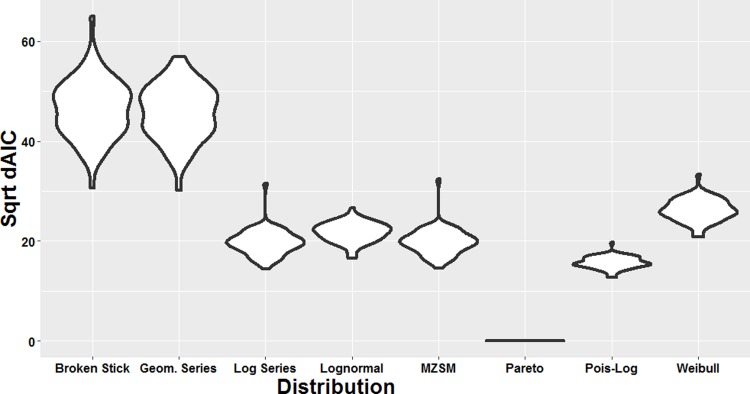
Violin plots of square root-transformed dAICc values for MLE fitted genus abundance distributions across all 60 samples. A value of 0 indicates the best-fitting distribution; higher dAICc values indicate poorer fits.

**Fig 6:**
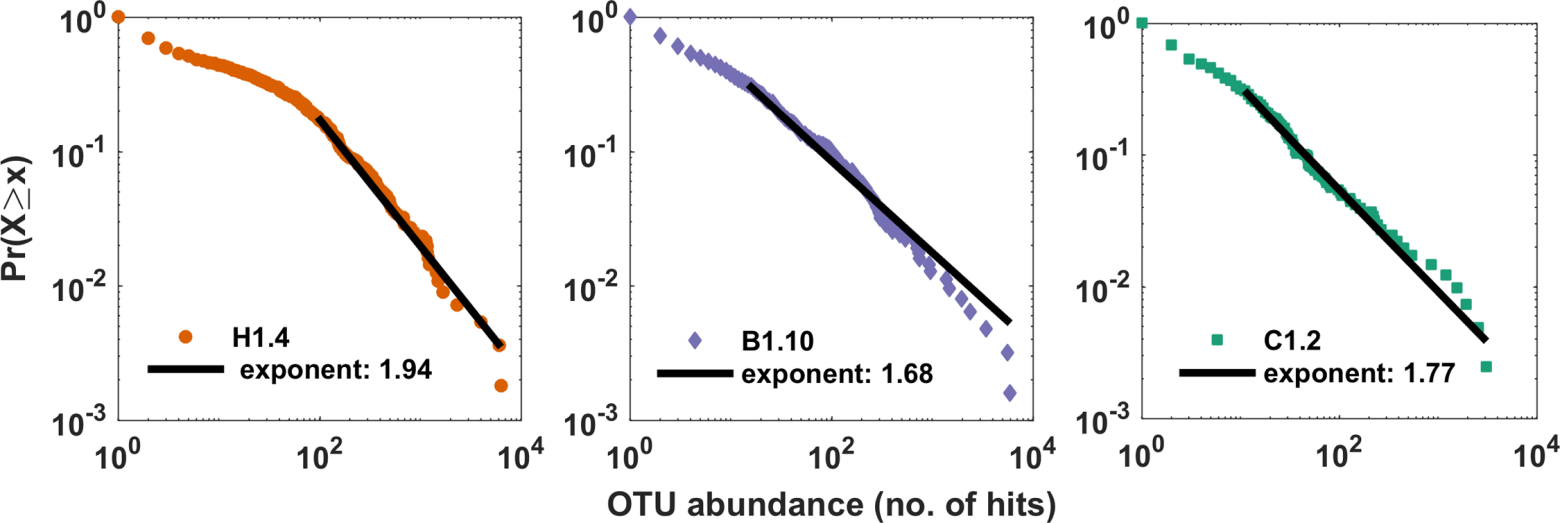
Representative power laws from sample genus abundance distributions for (A) hotspots (B) background and (C) coldspots.

Genus abundance distributions were created using all hotspot, coldspot and background regions (Figs 5 and 6). Using MLE to apply abundance distribution model fits to the taxonomic data from each 1 μl subsample at the genus-level, the Pareto (power law) distribution was clearly identified as the top ranked distribution for all samples, displaying the best (least) AICc values and Aikake weights of ~1 (Fig 5, S1 Table). After truncation for optimal model fit, and recalculation of the shape parameter, 58 of the 60 samples passed the goodness of fit test, i.e. the null hypothesis that the data was drawn from a power law distribution was accepted (5% significance threshold) (Fig 6, S2 Table). The 2 samples not fitting a power law were both from hotspots, and excluded from calculation of the mean hotspot power exponent (S2 Table). The exponent value of -2.7 from the coldspot region was identified as an outlier by Grubb’s test (p-value <1E-10, R 3.7.3) and also excluded from mean value calculations. The mean exponent for the hotspots, -1.90, was significantly lower than the means for the coldspots (-1.77) and background (-1.78) (1-way ANOVA, MATLAB R2017b; the multiple comparison p-values for coldspots and background were 2.0E-5 and 2.1E-5 respectively).

## Discussion

Here we report heterogeneity between the taxonomic profiles of 1 μl subsamples taken from 300 μl hotspots, coldspots and background regions, hence supporting our hypothesis (Figs 3 and S3). This heterogeneity was most extreme within hotspot subsamples and was due to the presence of hotspots within hotspots, here termed ‘micropatches’. These micropatches were 1 μl hotspot subsamples characterised by heightened relative abundance of specific individual genera, whilst the remaining hotspot subsamples showed consistent relative abundances of all genera. Consistent relative abundances of genera common to most subsamples may indicate balanced succession and exploitation, where all bacterial genera have the ability to take advantage of growth inducing signals such as nutrient patches [3, 64]. Whereas, exclusive dominance of specific genera may indicate specialist responses to specific localised environmental conditions [38, 39, 65].

Whether considering the few abundant taxa via Bray-Curtis dissimilarity (Fig 4A) or the many rare taxa via Sorensen distance metric (Fig 4B), distinct separation was observed between hotspot, coldspot and background samples as well as between sample wells of the same sample type. This suggests 300 μl microbial patches have taxonomically distinct groupings, which appear to remain coherent for extended periods. The highest dissimilarity and dispersion, as revealed by SIMPER, PERMdisp and MDS analysis, was between the hotspot subsamples, which is indicative of the higher taxonomic heterogeneity observed between these subsamples, indicating distinct community structure at the 1 μl scale (Fig 3). The taxonomic heterogeneity observed within 1 μl subsamples here is consistent with the previous findings of Long and Azam [9] who demonstrated heterogeneous species richness and composition across 1 μl seawater samples. In addition, Meckenstock [40] found heterogeneity within the OTU profiles of 1 μl samples from oil-confined, ancient water droplets. Patterns in the relative abundance of Proteobacteria, Firmicutes and Bacteroidetes were principally responsible for the heterogeneous composition of communities between hotspot subsamples indicating these phylogenetic groups play key roles in microbial microenvironment structure at the 1 μl scale (Figs 3A and S3A). Micropatches of Proteobacteria abundance is consistent with previous observations in freshwater systems, where a high incidence of Proteobacteria attachment to suspended particles has been demonstrated [66, 67]. Proteobacteria represent 20–50% total bacterial abundance within suspended river particles and biofilms, whilst their abundance is much lower in free-living bacterial communities [66, 67], therefore suggesting micropatches are due to particulate matter that leads to heightened abundance of specific taxa. *Pseudomonas* was the primary genus responsible for this elevated Proteobacteria abundance in one of the H2 hotspot subsamples (Fig 3A). As *Pseudomonas* is common within aquatic environments, and can indicate zoonotic or anthropogenic inputs [67, 68], its elevated abundance could correlate to particulate matter influx from the houseboats and cattle farming lands that surround the sampling site. In the individual micropatch of hotspot H1 that contained an elevated abundance of Proteobacteria, *Brevundimonas* was the primary genus present (Fig 3A). As *Brevundimonas* is often associated with high levels of organic compounds, particulate matter from cellular components may explain its elevated abundance [67, 68]. The elevated abundance of Firmicutes occurred in micropatches from hotspot H1 (S1A Fig). Firmicutes are abundant within sediment environments [69] but can also serve as important faecal-indicators in environmental water systems [70], again suggesting the micropatches are due to particles, in this instance, faecal or sediment-associated. *Lachnospiraceae incertae sedis* had elevated abundance in a micropatch from hotspot H1, whilst *Parasporobacterium* had elevated abundance in several micropatches from hotspot H1 (Fig 3A). *Lachnospiraceae incertae sedis* is anaerobic and involved in rumen biohydrogenation, therefore its abundance may be due to the presence of faecal particles from ruminant animals, which corresponds to the surrounding agricultural farming lands [71]. *Parasporobacterium* is an anaerobic bacterium typically isolated from freshwater benthos, therefore its heightened relative abundance may indicate resuspension of sediment into the water column [72]. As the river sampled is characterised by highly regulated flows and turbid waters, shearing of biofilms on the sediment-water interface is likely to occur. Lastly, elevated Bacteroidetes relative abundance in the micropatch hotspot subsample H3.4 (S1A Fig) was primarily driven by increases in the relative occurrence of *Bacteroides* whilst the most abundant Bacteroidetes genus within the remaining hotspot subsamples was *Flexibacter* (Fig 3A). As some Bacteroidetes are anaerobic bacteria responsible for specialised degradation of complex macro-molecules, they are typically found on suspended particles or in the anoxic regions of microbial biofilms as these contain refractory materials [66, 73]. Therefore, the heightened Bacteroidetes abundance may again, indicate the sampling of organic particles, such as river snow, or the shedding, detachment or disruption of cells from a biofilm containing a high concentration of this particular phylum [66, 74, 75].

Micropatches were due specifically to *Pseudomonas*, *Parasporobacterium*, *Lachnospiraceae incertae sedis* and *Bacteroides*, as they exhibited largely varying relative abundance proportions between subsamples, with heightened relative abundance within individual hotspot subsamples (Fig 3). Removal of these genera from the community composition yielded little difference to the similarity within and between sample types (S4 and S5 Tables), indicating these individual genera were not solely responsible for the heterogeneity between and within sample regions. This confirms micropatches are not due to the enrichment of a single genus, rather they are influenced by the type, combination and abundance of genera present, and therefore implies inter-organism interactions are more complex than the simple enrichment of a single genus. Furthermore, the removal of micropatch hotspot subsamples led to an increase in similarity between and within sample regions confirming the largest heterogeneity existed within the single 1 μl micropatch subsamples from hotspots. Hotspots remained the most heterogeneous of the sample regions despite removing the most heterogeneous subsamples from each hotspot sample (S4 and S5 Tables), confirming that hotspots are taxonomically distinct from coldspots and background regions. Therefore hotspots, coldspots and background regions are distinct taxonomic microenvironments with heterogeneity present within each of these sample types down to the 1 μl scale. Homogeneity was also observed within the hotspot, coldspot and background subsamples (Figs 3 and S3). After the removal of the micropatch hotspot subsamples, *Geothrix*, *Nocardioides*, *Flexibacter*, *Austwickia* and *Chryseoglobus*, were identified as the main drivers for similarity between subsamples as they had abundances that were common amongst subsamples. This indicates a common taxonomic community exists amongst subsamples from hotspot, coldspot and background regions. The abundant common taxonomic community is likely to be homogeneous in absolute abundance, but the rarer organisms in this community will increase in relative abundance within hotspot regions therefore causing the shifts observed in the most abundant genera between hotspot subsamples. For this to be possible, according to community assembly theory, these less abundant species can avoid local extinction and out compete other more abundant species if they are able to have a competitive advantage, become replenished from surrounding sources or access a different ecological niche [76].

Previously discrimination of microbial hotspots and coldspots had remained largely qualitative, with the former being primarily defined as abundance regions that are ‘elevated above’ or ‘exceed’ background variation across one or two sampling points [7, 34, 35]. Dann *et al*. [4] provided a quantitative method for discriminating hotspot, coldspot and background abundance values via rank abundance analysis, with sample values being separated based on their slope and line of best fit [4, 21, 37]. Here we expand on this analysis by using Chow tests to show significant structural breaks within these rank abundance distributions (Figs 2 and S1, Table 1). Background values were best described by a linear model, whereas hotspot and coldspot values had a nonlinear model–power law–best-fit (Fig 2, Table 1). It has previously been shown that random noise appears as linear [77], which indicates here that the background abundance values are due to random processes, such as has been observed previously in fractional Brownian motions [77], with no one sample value being more likely to be more common than any other sample value. A linear or random trend can be indicative of equilibrated additive and reductive processes [4]. Such additive processes in bacterial and viral communities could be reproduction or aggregation, whilst reductive processes could relate to grazing, decay or lysis events. Conversely, the hotspot and coldspot samples were identified via a clear structural discontinuity between the characteristic behaviours expected in cases of randomness (i.e. the background values), leading to a nonlinear power law best-fit for these sample values (Fig 2). Power law trends indicate nonrandomness and thus for the hotspots may indicate a favouring towards additive processes, whilst reductive processes may be favoured for the coldspots, resulting in a non-equilibrated state when compared to the background samples [4, 21, 77]. By using these rank abundance graphs, two linear trends were identified within the background region, therefore creating four distinct sections in the rank abundance profile (Fig 2, Table 1). Two linear trends have been observed previously within background values and were attributed to the presence of different randomisation processes [4, 21]. For instance, one linear trend may indicate an equilibrium between grazing and aggregation, whilst the second linear trend may indicate an equilibrium between reproduction and lysis. In order to distinguish between these randomisation processes experimental work is necessary.

Taxonomic distributions within the 1 μl subsamples from hotspot, coldspot and background samples revealed a power law best-fit for all sample types (Fig 5), with hotspots exhibiting significantly steeper mean exponent values when compared to the coldspots (Fig 6, S2 Table). These findings are in line with previous work by Mitchell [78] and Seymour et al. [7], which showed exponent values at and below −1 indicate high levels of structural complexity with considerable self-organisation and strong patches [78]. Here, this high level of structural complexity was observed as distinct taxonomic partitioning and high levels of taxonomic heterogeneity between 1 μl subsamples, with this being most extreme for hotspot samples (Figs 3 and 4).

## Conclusion

Here we report genetic heterogeneity among 1 μl subsamples retrieved from 300 μl microbial hotspots, coldspots and background regions that were determined by bacterial abundance patterns. This heterogeneity was typically driven by heightened relative abundance of specific genera, with this pattern most apparent within hotspot subsamples. Heterogeneity between subsamples revealed hotspot regions were characterised by either consistent abundances in genera common to most subsamples or the dominance of specific individual genera, here termed ‘micropatches’. These micropatches revealed a complex internal structure within hotspots. The genera exhibiting heightened abundance generally contained members of the Firmicutes, Proteobacteria and Bacteroidetes, implying the ability of these groups to exploit heterogeneous microenvironments and due to the particle-association of these phyla and respective genera, indicated particulate matter may be causing these distinct micropatches.

Here we expand on previous rank abundance discrimination of microbial abundance hotspots and coldspots by showing distinct and significant structural breaks occur between a linear background and hotspot and coldspot values that follow a power law trend. Taxonomic distributions were best explained by a power law, with hotspots characterised by steeper exponent values than the background and coldspots, owing to their higher level of structural complexity. We present this robust quantitative method for use in future studies on patch dynamics.

Previously, microscale heterogeneity studies have focussed on numerical differences [4, 7, 11, 12, 21, 48] and here we extend the work to include taxonomic gradients by showing that across a few millilitres there are strong and extensive taxonomic changes. As microbial hotspots and coldspots are important microenvironments for nutrient exchange and cellular interactions, understanding their taxonomic makeup will aid further understanding in environmental heterogeneity, microbial diversity and ecosystem function.

## Supporting information

S1 FigF-statistics and change points in the total prokaryote rank-abundance profile resulting from the ${\text{sup}}_{F}$ criterion for optimal distribution breaks between **(A)** hotspots-background **(B)** background-coldspot and **(C)** within background data.(TIF)

S2 Fig Normal QQ plots of the residuals (top) and residuals vs fitted (bottom) scatterplots for the chosen rank-abundance models for (L-R): hotspots; background (1); background (2); and coldspots. (TIF)

S3 Fig Representative flow cytometric biparametric cytogram of SYBR Green fluorescence vs. side scatter (size) showing the presence of two prokaryotic (LDNA and HDNA) and two viral (V1 and V2) subpopulations. (TIF)

S4 Fig Two-dimensional contour plots showing the presence of hotspots and coldspots in **A** LDNA, **B** HDNA, **C** V1, **D** V2 and **E** Total virus. Faint gridlines indicate sampling interval. Minimum contour value of ≥ 10000 chosen. Solid red regions indicate sample points higher than the maximum contour level selected. Solid white point indicates empty sample well. Colour intensity scale in particles ml^-1^.
(TIF)

S5 Fig Phylogenetic heatmaps of **A** hotspot, **B** coldspots and **C** background subsamples showing the heightened abundance of Firmicutes, Proteobacteria and Bacteroidetes. For clarity, only phyla representing > 2% average abundance are shown. OTUs determined via RDP Classifier within the UPARSE pipeline.
(TIF)

S1 File Viral abundance. (DOCX)

S1 Table Maximum likelihood parameter estimates and AICc ranks and weights for genus abundance distributions across all samples. (DOCX)

S2 Table MLE-calculated optimal Pareto (power) law truncation point, estimated best-fit exponent, and goodness of fit test information for genus abundance distributions across all samples. (DOCX)

S3 Table Total prokaryotic abundances determined via flow cytometry. (DOCX)

S4 Table SIMPER similarity comparisons between subsamples with no removal and removal of genera with heightened relative abundance. (DOCX)

S5 Table SIMPER similarity comparisons between samples with no removal and removal of genera with heightened relative abundance. (DOCX)
