# Supplementary figures and images for: Microbial micropatches within microbial hotspots
Source: PLoS One. 2018 May 22;13(5):e0197224. doi: 10.1371/journal.pone.0197224 (PMC5963804; doi:10.1371/journal.pone.0197224)

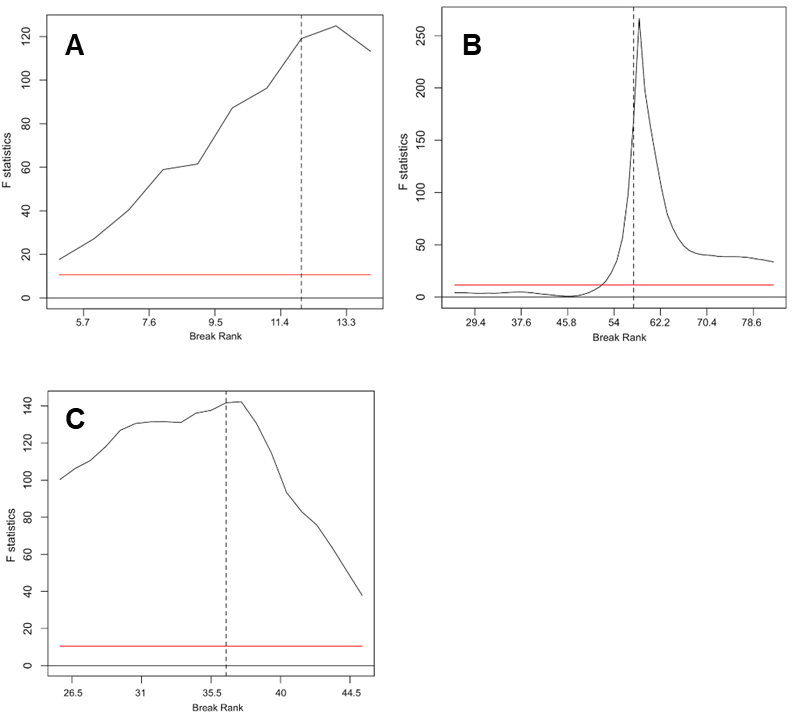

Supplement: S1 Fig — F-statistics and change points in the total prokaryote rank-abundance profile resulting from the supF criterion for optimal distribution breaks between (A) hotspots-background (B) background-coldspot and (C) within background data. (TIF) [file pone.0197224.s001.tif]

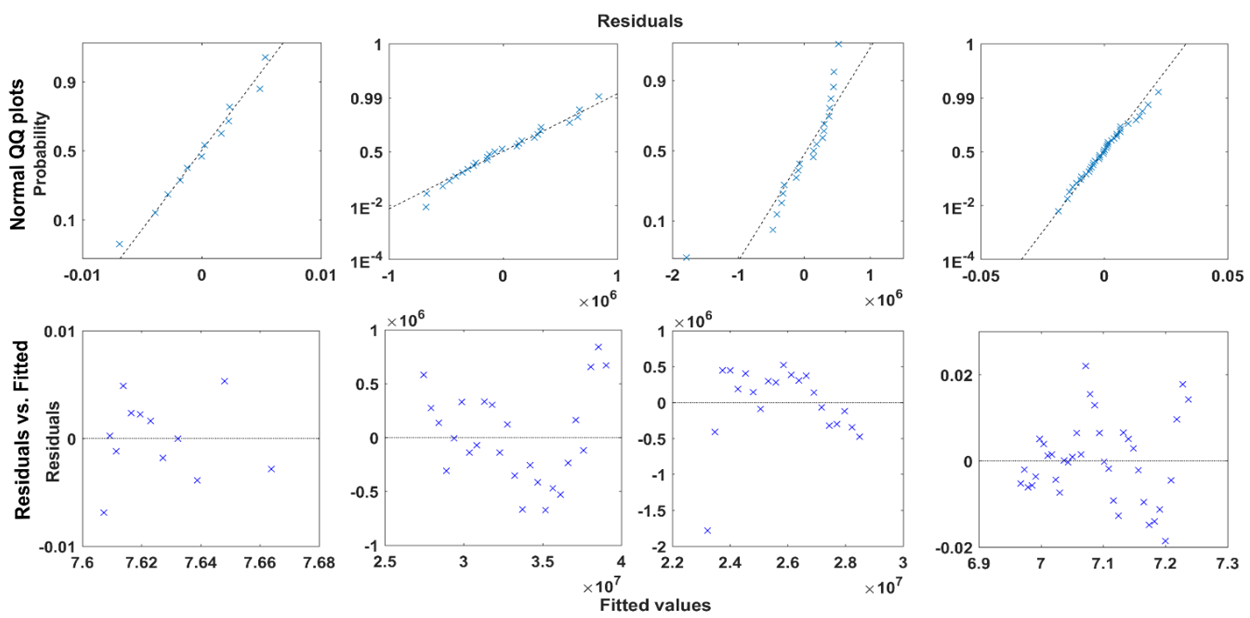

Supplement: S2 Fig — (TIF) [file pone.0197224.s002.tif]

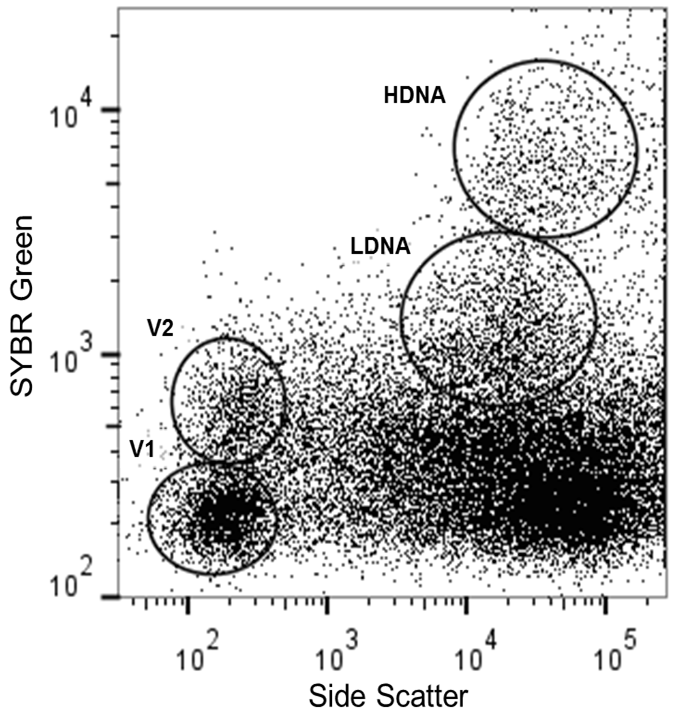

Supplement: S3 Fig — (TIF) [file pone.0197224.s003.tif]

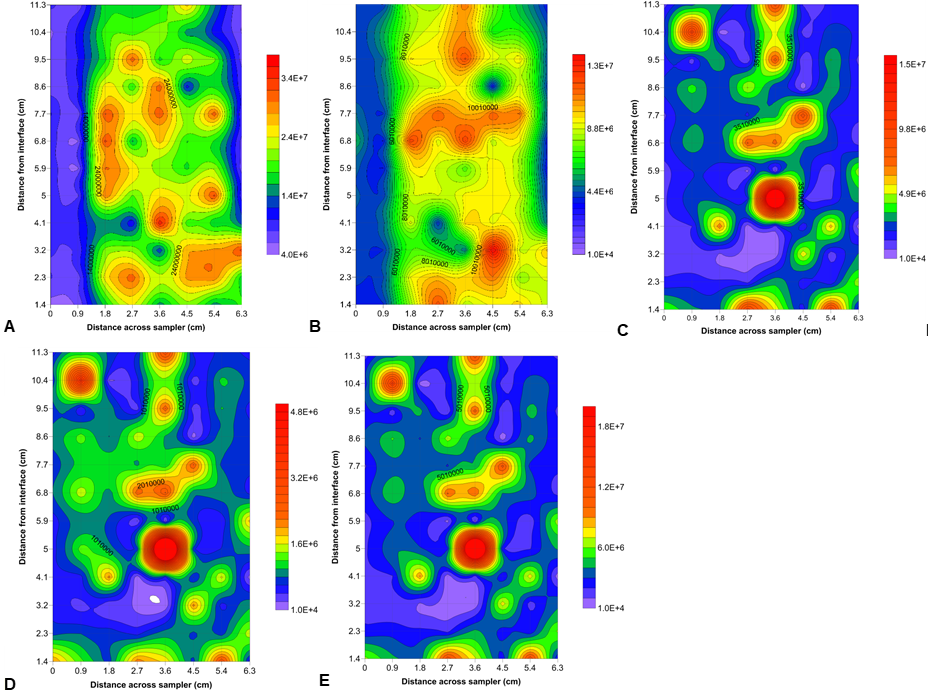

Supplement: S4 Fig — Two-dimensional contour plots showing the presence of hotspots and coldspots in A LDNA, B HDNA, C V1, D V2 and E Total virus. Faint gridlines indicate sampling interval. Minimum contour value of ≥ 10000 chosen. Solid red regions indicate sample points higher than the maximum contour level selected. Solid white point indicates empty sample well. Colour intensity scale in particles ml-1. (TIF) [file pone.0197224.s004.tif]

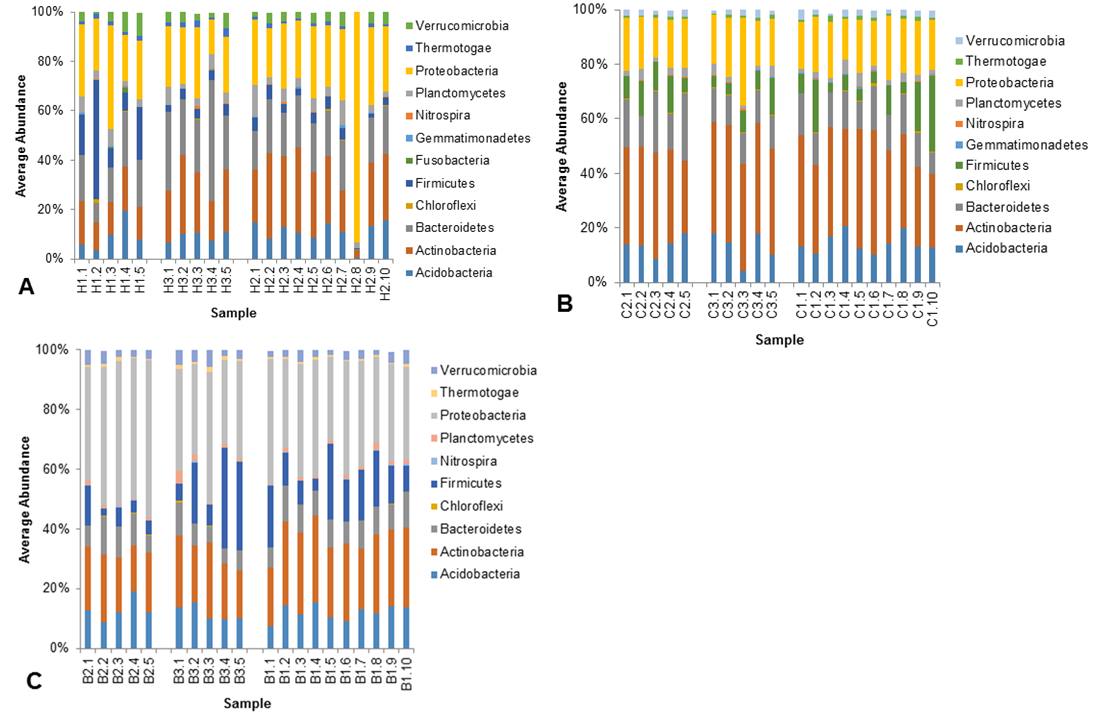

Supplement: S5 Fig — Phylogenetic heatmaps of A hotspot, B coldspots and C background subsamples showing the heightened abundance of Firmicutes, Proteobacteria and Bacteroidetes. For clarity, only phyla representing > 2% average abundance are shown. OTUs determined via RDP Classifier within the UPARSE pipeline. (TIF) [file pone.0197224.s005.tif]
