# Supplementary material for: Microbial micropatches within microbial hotspots
Source: PLoS One. 2018 May 22;13(5):e0197224. doi: 10.1371/journal.pone.0197224 (PMC5963804; doi:10.1371/journal.pone.0197224)
Supplement: S1 File — (DOCX) [file pone.0197224.s006.docx]

**Viral Abundance**

Flow cytometric analysis revealed 2 viral (V1 and V2) subpopulations. Viral abundances ranged from 2.5 × 10^5^ to 2.4 × 10^7^ particles ml^−1^ (mean = 2.9 × 10^6^, SD = 3.2 × 10^6^, n = 95) for the V1 subpopulation, from 4.5 × 10^4^ to 8.6 × 10^6^ particles ml^−1^ (mean = 1.0 × 10^6^, SD = 1.1 × 10^6^, n = 95) for the V2 subpopulation and from 2.9 × 10^5^ to 3.3 × 10^7^ particles ml^−1^ (mean = 4.0 × 10^6^, SD = 4.2 × 10^6^, n = 95) for total viruses. Hotspots and coldspots were also present in the viral distributions (S4 Fig.) with V1 having a maximum hotspot of 2.4 × 10^7^ particles ml^−1^ and a minimum coldspot of 2.5 × 10^5^ particles ml^−1^, resulting in an overall 97.5-fold change in abundance over the sampling area. V2 had a maximum hotspot of 8.8 × 10^6^ particles ml^−1^ and a minimum coldspot of 4.5 × 10^4^ particles ml^−1^ resulting in an overall 195.4-fold change in abundance over the sampling area. From one sampling well to the next, the largest change in abundance for V1 was 84.2- fold, V2 was 115-fold, and total viruses was 90.6-fold per 0.9 cm.
