# Supplementary material for: Microbial micropatches within microbial hotspots
Source: PLoS One. 2018 May 22;13(5):e0197224. doi: 10.1371/journal.pone.0197224 (PMC5963804; doi:10.1371/journal.pone.0197224)
Supplement: S1 Table — (DOCX) [file pone.0197224.s007.docx]

| **S1 Table. Maximum likelihood parameter estimates and AICc ranks and weights for genus abundance distributions across all samples.** | | | | | | | | | | | | | | | | | | | | | | | | | | | | | | | | | | | | | | | | | | | | | | | | | | | | | | | | | | | | |
| --- | --- | --- | --- | --- | --- | --- | --- | --- | --- | --- | --- | --- | --- | --- | --- | --- | --- | --- | --- | --- | --- | --- | --- | --- | --- | --- | --- | --- | --- | --- | --- | --- | --- | --- | --- | --- | --- | --- | --- | --- | --- | --- | --- | --- | --- | --- | --- | --- | --- | --- | --- | --- | --- | --- | --- | --- | --- | --- | --- | --- |
| **Model** | H1.1 | H1.2 | H1.3 | H1.4 | H1.5 | H1.6 | H1.7 | H1.8 | H1.9 | H1.10 | H2.1 | H2.2 | H2.3 | H2.4 | H2.5 | H3.1 | H3.2 | H3.3 | H3.4 | H3.5 | C1.1 | C1.2 | C1.3 | C1.4 | C1.5 | C1.6 | C1.7 | C1.8 | C1.9 | C1.10 | C2.1 | C2.2 | C2.3 | C2.4 | C2.5 | C3.1 | C3.2 | C3.3 | C3.4 | C3.5 | B1.1 | B1.2 | B1.3 | B1.4 | B1.5 | B1.6 | B1.7 | B1.8 | B1.9 | B1.10 | B2.1 | B2.2 | B2.3 | B2.4 | B2.5 | B3.1 | B3.2 | B3.3 | B3.4 | B3.5 |
| Broken Stick N | 77524.00 | 74376.00 | 53862.00 | 58649.00 | 52171.00 | 38357.00 | 115285.00 | 75840.00 | 60673.00 | 75728.00 | 114499.00 | 46545.00 | 78882.00 | 98576.00 | 98963.00 | 102395.00 | 55989.00 | 108025.00 | 146072.00 | 104977.00 | 34548.00 | 18612.00 | 52884.00 | 47636.00 | 28143.00 | 31294.00 | 35560.00 | 29726.00 | 49343.00 | 35054.00 | 63451.00 | 35690.00 | 46502.00 | 34271.00 | 50443.00 | 49736.00 | 9373.00 | 38866.00 | 66713.00 | 121978.00 | 21923.00 | 53874.00 | 99216.00 | 18344.00 | 73859.00 | 33994.00 | 48250.00 | 81574.00 | 42040.00 | 43603.00 | 21210.00 | 15399.00 | 27376.00 | 31150.00 | 24112.00 | 65631.00 | 61996.00 | 27283.00 | 49771.00 | 48814.00 |
| Broken Stick S | 568.00 | 550.00 | 509.00 | 555.00 | 522.00 | 468.00 | 635.00 | 423.00 | 537.00 | 577.00 | 673.00 | 471.00 | 531.00 | 653.00 | 663.00 | 644.00 | 505.00 | 567.00 | 661.00 | 619.00 | 508.00 | 407.00 | 544.00 | 497.00 | 402.00 | 472.00 | 471.00 | 485.00 | 550.00 | 481.00 | 603.00 | 460.00 | 569.00 | 469.00 | 572.00 | 557.00 | 331.00 | 426.00 | 647.00 | 684.00 | 454.00 | 644.00 | 716.00 | 402.00 | 605.00 | 558.00 | 564.00 | 662.00 | 532.00 | 625.00 | 420.00 | 391.00 | 427.00 | 483.00 | 393.00 | 661.00 | 573.00 | 442.00 | 495.00 | 538.00 |
| Broken Stick Log Lik | -3373.75 | -3260.61 | -2892.69 | -3150.93 | -2933.08 | -2539.81 | -3948.97 | -2902.96 | -3086.57 | -3403.45 | -4140.32 | -2674.80 | -3201.91 | -3947.17 | -3995.84 | -3919.32 | -2891.93 | -3552.68 | -4253.37 | -3806.34 | -2665.28 | -1978.26 | -3053.15 | -2785.05 | -2122.84 | -2467.55 | -2521.23 | -2498.30 | -3040.68 | -2566.49 | -3428.35 | -2474.72 | -3091.06 | -2493.92 | -3152.11 | -3078.66 | -1447.14 | -2354.72 | -3669.29 | -4247.23 | -2237.70 | -3515.29 | -4268.23 | -1952.86 | -3533.58 | -2866.02 | -3091.43 | -3867.59 | -2875.06 | -3294.61 | -2087.21 | -1854.39 | -2235.53 | -2532.87 | -2036.23 | -3722.68 | -3279.01 | -2283.20 | -2817.99 | -2997.53 |
| Geom Series k | 0.01 | 0.01 | 0.01 | 0.01 | 0.01 | 0.01 | 0.01 | 0.06 | 0.01 | 0.01 | 0.01 | 0.01 | 0.01 | 0.01 | 0.01 | 0.01 | 0.01 | 0.01 | 0.00 | 0.01 | 0.01 | 0.02 | 0.01 | 0.01 | 0.01 | 0.02 | 0.01 | 0.02 | 0.01 | 0.01 | 0.01 | 0.01 | 0.01 | 0.01 | 0.01 | 0.01 | 0.04 | 0.01 | 0.01 | 0.01 | 0.02 | 0.01 | 0.01 | 0.02 | 0.01 | 0.02 | 0.01 | 0.01 | 0.01 | 0.01 | 0.02 | 0.03 | 0.02 | 0.02 | 0.02 | 0.01 | 0.01 | 0.02 | 0.01 | 0.01 |
| Geom Series Log Lik | -3358.33 | -3246.80 | -2879.41 | -3138.86 | -2922.99 | -2527.25 | -3936.22 | na | -3073.15 | -3388.86 | -4127.93 | -2632.06 | -3184.71 | -3926.94 | -3979.57 | -3906.34 | -2880.43 | -3542.12 | -4227.65 | -3794.74 | -2647.82 | -1958.38 | -3031.03 | -2762.09 | -2107.05 | -2448.09 | -2504.53 | -2477.10 | -3020.07 | -2540.59 | -3407.76 | -2458.67 | -3071.02 | -2478.47 | -3131.00 | -3055.87 | -1431.77 | -2346.38 | -3643.22 | -4227.69 | -2209.51 | -3490.93 | -4244.27 | -1933.45 | -3509.35 | -2846.54 | -3069.98 | -3846.17 | -2853.32 | -3273.71 | -2063.04 | -1822.27 | -2200.24 | -2491.68 | -2007.63 | -3696.97 | -3254.24 | -2260.64 | -2774.79 | -2960.28 |
| Log Series alpha | 77524.00 | 74376.00 | 53862.00 | 58649.00 | 52171.00 | 38357.00 | 115285.00 | 75840.00 | 60673.00 | 75728.00 | 114499.00 | 46545.00 | 78882.00 | 98576.00 | 98963.00 | 102395.00 | 55989.00 | 108025.00 | 146072.00 | 104977.00 | 34548.00 | 18612.00 | 52884.00 | 47636.00 | 28143.00 | 31294.00 | 35560.00 | 29726.00 | 49343.00 | 35054.00 | 63451.00 | 35690.00 | 46502.00 | 34271.00 | 50443.00 | 49736.00 | 9373.00 | 38866.00 | 66713.00 | 121978.00 | 21923.00 | 53874.00 | 99216.00 | 18344.00 | 73859.00 | 33994.00 | 48250.00 | 81574.00 | 42040.00 | 43603.00 | 21210.00 | 15399.00 | 27376.00 | 31150.00 | 24112.00 | 65631.00 | 61996.00 | 27283.00 | 49771.00 | 48814.00 |
| Log Series Log Lik | -2316.12 | -2330.93 | -2125.58 | -2354.21 | -2202.97 | -1938.23 | -2859.95 | -1286.45 | -2276.20 | -2483.85 | -2951.79 | -1810.41 | -2221.47 | -2799.83 | -2895.46 | -2813.03 | -2115.65 | -2378.23 | -2995.46 | -2748.57 | -1880.40 | -1422.17 | -2049.92 | -1901.77 | -1512.68 | -1746.12 | -1741.35 | -1775.53 | -2094.88 | -1809.29 | -2349.84 | -1691.46 | -2124.39 | -1752.97 | -2197.69 | -2139.74 | -1101.66 | -1640.40 | -2593.74 | -2863.92 | -1598.50 | -2462.90 | -2928.81 | -1418.55 | -2361.14 | -2125.57 | -2151.09 | -2642.60 | -1983.59 | -2415.66 | -1456.76 | -1323.67 | -1551.38 | -1761.60 | -1483.88 | -2596.06 | -2287.55 | -1599.13 | -1896.17 | -1993.25 |
| MSZM theta | 77524.00 | 74376.00 | 53862.00 | 58649.00 | 52171.00 | 38357.00 | 115285.00 | 75840.00 | 60673.00 | 75728.00 | 114499.00 | 46545.00 | 78882.00 | 98576.00 | 98963.00 | 102395.00 | 55989.00 | 108025.00 | 146072.00 | 104977.00 | 34548.00 | 18612.00 | 52884.00 | 47636.00 | 28143.00 | 31294.00 | 35560.00 | 29726.00 | 49343.00 | 35054.00 | 63451.00 | 35690.00 | 46502.00 | 34271.00 | 50443.00 | 49736.00 | 9373.00 | 38866.00 | 66713.00 | 121978.00 | 21923.00 | 53874.00 | 99216.00 | 18344.00 | 73859.00 | 33994.00 | 48250.00 | 81574.00 | 42040.00 | 43603.00 | 21210.00 | 15399.00 | 27376.00 | 31150.00 | 24112.00 | 65631.00 | 61996.00 | 27283.00 | 49771.00 | 48814.00 |
| MSZM Log Lik | -2317.67 | -2332.27 | -2126.86 | -2355.28 | -2203.76 | -1939.42 | -2861.10 | -1315.02 | -2277.49 | -2485.29 | -2952.88 | -1815.98 | -2223.28 | -2802.02 | -2897.09 | -2814.21 | -2116.68 | -2379.11 | -2998.29 | -2749.56 | -1882.29 | -1424.55 | -2052.50 | -1904.50 | -1514.42 | -1748.38 | -1743.17 | -1778.05 | -2097.24 | -1812.54 | -2352.15 | -1693.19 | -2126.64 | -1754.60 | -2200.10 | -2142.42 | -1103.22 | -1640.99 | -2596.83 | -2865.98 | -1602.21 | -2465.76 | -2931.50 | -1420.86 | -2363.95 | -2127.71 | -2153.56 | -2644.97 | -1986.14 | -2417.98 | -1459.84 | -1328.17 | -1556.16 | -1767.24 | -1487.66 | -2599.09 | -2290.48 | -1601.91 | -1901.79 | -1998.07 |
| Pareto shape | 0.50 | 0.46 | 0.47 | 0.45 | 0.45 | 0.46 | 0.42 | 1.07 | 0.45 | 0.45 | 0.44 | 0.55 | 0.48 | 0.45 | 0.44 | 0.44 | 0.46 | 0.48 | 0.42 | 0.43 | 0.57 | 0.62 | 0.57 | 0.55 | 0.56 | 0.57 | 0.58 | 0.58 | 0.55 | 0.56 | 0.54 | 0.59 | 0.57 | 0.57 | 0.54 | 0.54 | 0.65 | 0.54 | 0.50 | 0.48 | 0.62 | 0.55 | 0.50 | 0.61 | 0.54 | 0.54 | 0.55 | 0.52 | 0.57 | 0.53 | 0.64 | 0.66 | 0.59 | 0.59 | 0.55 | 0.52 | 0.51 | 0.60 | 0.55 | 0.59 |
| Pareto scale | 1.00 | 1.00 | 1.00 | 1.00 | 1.00 | 1.00 | 1.00 | 1.00 | 1.00 | 1.00 | 1.00 | 1.00 | 1.00 | 1.00 | 1.00 | 1.00 | 1.00 | 1.00 | 1.00 | 1.00 | 1.00 | 1.00 | 1.00 | 1.00 | 1.00 | 1.00 | 1.00 | 1.00 | 1.00 | 1.00 | 1.00 | 1.00 | 1.00 | 1.00 | 1.00 | 1.00 | 1.00 | 1.00 | 1.00 | 1.00 | 1.00 | 1.00 | 1.00 | 1.00 | 1.00 | 1.00 | 1.00 | 1.00 | 1.00 | 1.00 | 1.00 | 1.00 | 1.00 | 1.00 | 1.00 | 1.00 | 1.00 | 1.00 | 1.00 | 1.00 |
| Pareto Log Lik | -2105.15 | -2169.99 | -1989.31 | -2222.57 | -2080.55 | -1832.84 | -2709.72 | -788.64 | -2140.14 | -2336.14 | -2767.43 | -1612.74 | -2041.91 | -2608.37 | -2726.13 | -2640.42 | -1978.36 | -2160.01 | -2821.51 | -2593.93 | -1674.13 | -1251.07 | -1800.77 | -1692.11 | -1360.37 | -1557.03 | -1536.14 | -1580.55 | -1866.34 | -1621.74 | -2105.16 | -1482.41 | -1876.96 | -1563.48 | -1972.85 | -1918.93 | -976.88 | -1471.42 | -2371.91 | -2607.54 | -1407.96 | -2211.87 | -2665.08 | -1258.35 | -2096.67 | -1948.35 | -1924.90 | -2382.96 | -1755.26 | -2213.96 | -1261.20 | -1149.56 | -1367.62 | -1556.97 | -1351.21 | -2347.57 | -2079.91 | -1409.39 | -1682.80 | -1736.24 |
| Pois-Log mu | -2.16 | -1.71 | -1.50 | -1.44 | -1.20 | -0.62 | -1.22 | -3.95 | -1.33 | -1.44 | -1.41 | -2.19 | -1.91 | -1.67 | -1.23 | -1.45 | -1.57 | -2.14 | -1.07 | -1.33 | -2.23 | -1.71 | -2.48 | -2.29 | -2.09 | -2.08 | -2.17 | -2.07 | -2.20 | -1.78 | -2.11 | -2.58 | -2.34 | -2.24 | -1.81 | -2.15 | -1.26 | -2.60 | -1.31 | -1.75 | -1.00 | -1.51 | -1.24 | -1.84 | -1.92 | -0.76 | -1.99 | -1.73 | -2.27 | -0.96 | -2.24 | -1.99 | -1.84 | -1.60 | -0.39 | -1.57 | -1.10 | -2.38 | -2.05 | -2.11 |
| Pois-Log sigma | 3.88 | 3.90 | 3.77 | 3.82 | 3.70 | 3.37 | 3.97 | 3.05 | 3.76 | 3.87 | 3.92 | 3.64 | 3.90 | 3.93 | 3.83 | 3.92 | 3.81 | 3.98 | 3.90 | 3.96 | 3.54 | 3.15 | 3.66 | 3.66 | 3.56 | 3.49 | 3.50 | 3.45 | 3.62 | 3.43 | 3.67 | 3.63 | 3.60 | 3.58 | 3.51 | 3.64 | 2.87 | 3.84 | 3.48 | 3.80 | 2.87 | 3.37 | 3.50 | 3.25 | 3.57 | 3.08 | 3.55 | 3.61 | 3.57 | 3.21 | 3.31 | 3.16 | 3.31 | 3.23 | 2.85 | 3.49 | 3.35 | 3.51 | 3.56 | 3.44 |
| Pois-Log Log Lik | -2244.46 | -2285.79 | -2090.50 | -2329.37 | -2177.36 | -1914.95 | -2824.23 | -984.85 | -2241.81 | -2447.38 | -2897.14 | -1728.89 | -2161.47 | -2744.60 | -2846.95 | -2765.32 | -2080.36 | -2303.49 | -2935.55 | -2710.42 | -1803.60 | -1356.85 | -1946.63 | -1817.97 | -1458.70 | -1674.87 | -1656.95 | -1702.74 | -2003.72 | -1735.46 | -2250.81 | -1609.20 | -2025.22 | -1682.14 | -2106.61 | -2055.17 | -1064.32 | -1591.31 | -2506.04 | -2755.72 | -1519.53 | -2358.33 | -2810.71 | -1361.71 | -2239.41 | -2064.28 | -2060.43 | -2530.83 | -1891.75 | -2344.08 | -1377.33 | -1257.80 | -1474.81 | -1675.11 | -1431.15 | -2493.63 | -2197.57 | -1526.78 | -1802.49 | -1874.33 |
| Lognormal s | 2.01 | 2.17 | 2.15 | 2.21 | 2.20 | 2.15 | 2.39 | 0.93 | 2.20 | 2.24 | 2.29 | 1.82 | 2.10 | 2.20 | 2.29 | 2.28 | 2.15 | 2.08 | 2.40 | 2.34 | 1.74 | 1.60 | 1.75 | 1.81 | 1.80 | 1.74 | 1.72 | 1.72 | 1.80 | 1.79 | 1.87 | 1.69 | 1.74 | 1.77 | 1.84 | 1.84 | 1.53 | 1.84 | 1.98 | 2.08 | 1.62 | 1.83 | 2.02 | 1.64 | 1.85 | 1.87 | 1.82 | 1.94 | 1.74 | 1.90 | 1.56 | 1.52 | 1.68 | 1.70 | 1.83 | 1.91 | 1.96 | 1.67 | 1.81 | 1.70 |
| Lognormal omega | 2.22 | 2.21 | 2.10 | 2.15 | 2.09 | 1.97 | 2.31 | 1.41 | 2.11 | 2.19 | 2.25 | 1.94 | 2.22 | 2.25 | 2.18 | 2.25 | 2.13 | 2.37 | 2.26 | 2.30 | 1.86 | 1.65 | 1.97 | 1.97 | 1.88 | 1.83 | 1.84 | 1.80 | 1.93 | 1.82 | 1.98 | 1.92 | 1.90 | 1.89 | 1.89 | 1.95 | 1.55 | 2.21 | 1.94 | 2.13 | 1.61 | 1.83 | 1.97 | 1.70 | 1.91 | 1.78 | 1.89 | 1.97 | 1.88 | 1.82 | 1.68 | 1.61 | 1.74 | 1.72 | 1.71 | 1.91 | 1.89 | 1.81 | 1.88 | 1.80 |
| Lognormal Log Lik | -2399.59 | -2411.24 | -2192.11 | -2438.84 | -2272.49 | -1987.46 | -2953.27 | -1141.65 | -2343.33 | -2564.85 | -3039.86 | -1839.01 | -2292.79 | -2895.05 | -2973.42 | -2901.60 | -2185.07 | -2472.71 | -3060.32 | -2841.71 | -1920.93 | -1434.49 | -2092.31 | -1941.22 | -1547.78 | -1777.45 | -1764.39 | -1806.78 | -2134.66 | -1830.81 | -2392.16 | -1732.91 | -2165.37 | -1791.46 | -2227.59 | -2185.36 | -1120.71 | -1727.94 | -2627.85 | -2911.77 | -1595.79 | -2482.26 | -2946.89 | -1441.00 | -2370.56 | -2153.88 | -2183.29 | -2672.80 | -2017.14 | -2449.56 | -1468.01 | -1335.32 | -1559.78 | -1764.98 | -1488.41 | -2625.35 | -2299.32 | -1629.56 | -1909.40 | -1993.26 |
| Power s | 1.40 | 1.37 | 1.38 | 1.37 | 1.37 | 1.38 | 1.34 | 1.72 | 1.37 | 1.36 | 1.36 | 1.43 | 1.38 | 1.37 | 1.36 | 1.36 | 1.38 | 1.39 | 1.34 | 1.35 | 1.45 | 1.48 | 1.44 | 1.43 | 1.43 | 1.45 | 1.45 | 1.45 | 1.43 | 1.44 | 1.42 | 1.46 | 1.45 | 1.44 | 1.43 | 1.43 | 1.49 | 1.43 | 1.40 | 1.39 | 1.47 | 1.43 | 1.40 | 1.47 | 1.42 | 1.42 | 1.43 | 1.41 | 1.45 | 1.42 | 1.49 | 1.50 | 1.46 | 1.46 | 1.43 | 1.41 | 1.41 | 1.46 | 1.43 | 1.45 |
| Power Log Lik | -2240.99 | -2293.14 | -2104.43 | -2344.88 | -2196.16 | -1938.45 | -2840.40 | -975.62 | -2259.10 | -2461.81 | -2911.58 | -1734.94 | -2164.08 | -2752.67 | -2868.16 | -2778.80 | -2092.28 | -2291.76 | -2957.49 | -2723.84 | -1811.11 | -1368.60 | -1946.80 | -1821.80 | -1465.92 | -1684.18 | -1664.47 | -1712.79 | -2010.35 | -1748.50 | -2258.55 | -1609.23 | -2030.24 | -1688.49 | -2120.16 | -2062.55 | -1076.28 | -1580.97 | -2528.41 | -2766.36 | -1537.94 | -2378.43 | -2835.55 | -1372.40 | -2251.73 | -2090.27 | -2071.71 | -2546.15 | -1898.54 | -2370.59 | -1385.25 | -1267.40 | -1486.06 | -1690.09 | -1452.75 | -2512.79 | -2219.94 | -1532.54 | -1812.16 | -1884.36 |
| Weibull shape | 0.40 | 0.41 | 0.44 | 0.44 | 0.44 | 0.46 | 0.41 | 0.41 | 0.44 | 0.42 | 0.40 | 0.44 | 0.40 | 0.41 | 0.42 | 0.41 | 0.43 | 0.38 | 0.40 | 0.40 | 0.45 | 0.49 | 0.42 | 0.43 | 0.45 | 0.46 | 0.44 | 0.46 | 0.43 | 0.46 | 0.43 | 0.43 | 0.44 | 0.45 | 0.44 | 0.44 | 0.53 | 0.41 | 0.44 | 0.40 | 0.49 | 0.45 | 0.42 | 0.49 | 0.42 | 0.48 | 0.44 | 0.42 | 0.44 | 0.47 | 0.47 | 0.50 | 0.47 | 0.47 | 0.48 | 0.44 | 0.44 | 0.46 | 0.44 | 0.44 |
| Weibull scale | 24.58 | 28.40 | 25.97 | 28.18 | 27.14 | 24.24 | 36.98 | 5.96 | 27.43 | 29.91 | 32.52 | 17.62 | 26.74 | 29.87 | 31.15 | 31.96 | 26.62 | 28.72 | 36.21 | 35.01 | 15.68 | 12.23 | 16.85 | 17.75 | 16.73 | 15.42 | 15.29 | 14.81 | 17.35 | 15.98 | 18.88 | 15.55 | 16.14 | 16.28 | 17.61 | 17.99 | 10.70 | 20.81 | 20.59 | 25.41 | 12.16 | 16.85 | 21.94 | 12.92 | 18.08 | 16.77 | 17.13 | 20.33 | 15.87 | 17.80 | 11.92 | 10.98 | 13.81 | 13.78 | 15.66 | 18.93 | 19.66 | 14.25 | 16.82 | 14.66 |
| Weibull Log Lik | -2500.34 | -2497.38 | -2270.34 | -2514.63 | -2349.93 | -2058.91 | -3041.86 | -1344.03 | -2424.30 | -2649.55 | -3146.14 | -1935.74 | -2382.09 | -2995.61 | -3075.44 | -2998.31 | -2263.82 | -2573.41 | -3163.92 | -2932.54 | -2028.65 | -1530.54 | -2211.65 | -2044.61 | -1629.34 | -1878.44 | -1874.72 | -1911.16 | -2251.75 | -1933.51 | -2518.52 | -1833.13 | -2290.26 | -1890.12 | -2353.12 | -2297.64 | -1194.19 | -1799.88 | -2757.63 | -3050.48 | -1708.27 | -2625.41 | -3103.07 | -1529.69 | -2512.39 | -2265.13 | -2304.44 | -2816.71 | -2133.53 | -2573.98 | -1571.11 | -1427.94 | -1658.62 | -1875.82 | -1572.95 | -2766.18 | -2423.21 | -1725.38 | -2017.49 | -2126.76 |
| Broken Stick AIC | 6747.50 | 6521.23 | 5785.39 | 6301.86 | 5866.15 | 5079.63 | 7897.95 | 5805.93 | 6173.15 | 6806.90 | 8280.64 | 5349.60 | 6403.82 | 7894.34 | 7991.68 | 7838.64 | 5783.87 | 7105.37 | 8506.75 | 7612.67 | 5330.55 | 3956.52 | 6106.29 | 5570.09 | 4245.68 | 4935.11 | 5042.46 | 4996.61 | 6081.36 | 5132.98 | 6856.71 | 4949.43 | 6182.12 | 4987.84 | 6304.21 | 6157.31 | 2894.28 | 4709.43 | 7338.58 | 8494.46 | 4475.40 | 7030.58 | 8536.45 | 3905.72 | 7067.16 | 5732.04 | 6182.85 | 7735.18 | 5750.12 | 6589.22 | 4174.42 | 3708.79 | 4471.07 | 5065.74 | 4072.46 | 7445.37 | 6558.03 | 4566.41 | 5635.98 | 5995.06 |
| Broken Stick dAIC | 2533.17 | 2177.22 | 1802.74 | 1852.70 | 1701.04 | 1409.93 | 2474.48 | 4224.61 | 1888.83 | 2130.59 | 2741.77 | 2120.10 | 2315.99 | 2673.57 | 2535.39 | 2553.79 | 1823.13 | 2781.33 | 2859.71 | 2420.80 | 1978.27 | 1450.36 | 2500.73 | 2181.85 | 1520.91 | 1817.01 | 1966.14 | 1831.48 | 2344.65 | 1885.49 | 2642.36 | 1980.58 | 2424.17 | 1856.85 | 2354.50 | 2315.43 | 936.48 | 1762.56 | 2590.74 | 3275.36 | 1655.45 | 2602.83 | 3202.27 | 1384.99 | 2869.80 | 1831.33 | 2329.03 | 2965.25 | 2235.58 | 2157.28 | 1648.00 | 1405.64 | 1731.81 | 1947.78 | 1366.01 | 2746.21 | 2394.19 | 1743.60 | 2266.36 | 2518.56 |
| Broken Stick df | 0.00 | 0.00 | 0.00 | 0.00 | 0.00 | 0.00 | 0.00 | 0.00 | 0.00 | 0.00 | 0.00 | 0.00 | 0.00 | 0.00 | 0.00 | 0.00 | 0.00 | 0.00 | 0.00 | 0.00 | 0.00 | 0.00 | 0.00 | 0.00 | 0.00 | 0.00 | 0.00 | 0.00 | 0.00 | 0.00 | 0.00 | 0.00 | 0.00 | 0.00 | 0.00 | 0.00 | 0.00 | 0.00 | 0.00 | 0.00 | 0.00 | 0.00 | 0.00 | 0.00 | 0.00 | 0.00 | 0.00 | 0.00 | 0.00 | 0.00 | 0.00 | 0.00 | 0.00 | 0.00 | 0.00 | 0.00 | 0.00 | 0.00 | 0.00 | 0.00 |
| Broken Stick AIC weight | 0.00 | 0.00 | 0.00 | 0.00 | 0.00 | 0.00 | 0.00 | 0.00 | 0.00 | 0.00 | 0.00 | 0.00 | 0.00 | 0.00 | 0.00 | 0.00 | 0.00 | 0.00 | 0.00 | 0.00 | 0.00 | 1.144 | 0.00 | 0.00 | 0.00 | 0.00 | 0.00 | 0.00 | 0.00 | 0.00 | 0.00 | 0.00 | 0.00 | 0.00 | 0.00 | 0.00 | 0.00 | 0.00 | 0.00 | 0.00 | 0.00 | 0.00 | 0.00 | 0.00 | 0.00 | 0.00 | 0.00 | 0.00 | 0.00 | 0.00 | 0.00 | 0.00 | 0.00 | 0.00 | 0.00 | 0.00 | 0.00 | 0.00 | 0.00 | 0.00 |
| Geom Series AIC | 6718.66 | 6495.60 | 5760.83 | 6279.74 | 5847.98 | 5056.50 | 7874.45 | Inf | 6148.31 | 6779.73 | 8257.87 | 5266.13 | 6371.43 | 7855.88 | 7961.15 | 7814.68 | 5762.87 | 7086.25 | 8457.30 | 7591.49 | 5297.64 | 3918.76 | 6064.07 | 5526.18 | 4216.12 | 4898.18 | 5011.06 | 4956.21 | 6042.15 | 5083.18 | 6817.52 | 4919.34 | 6144.05 | 4958.94 | 6264.00 | 6113.74 | 2865.56 | 4694.77 | 7288.45 | 8457.38 | 4421.03 | 6983.87 | 8490.55 | 3868.90 | 7020.71 | 5695.09 | 6141.97 | 7694.36 | 5708.64 | 6549.42 | 4128.09 | 3646.56 | 4402.50 | 4985.37 | 4017.26 | 7395.95 | 6510.49 | 4523.28 | 5551.59 | 5922.57 |
| Geom Series dAIC | 2504.33 | 2151.59 | 1778.19 | 1830.57 | 1682.87 | 1386.80 | 2450.99 | Inf | 1864.00 | 2103.42 | 2719.00 | 2036.63 | 2283.60 | 2635.11 | 2504.86 | 2529.83 | 1802.14 | 2762.21 | 2810.26 | 2399.61 | 1945.35 | 1412.60 | 2458.51 | 2137.94 | 1491.35 | 1780.09 | 1934.75 | 1791.09 | 2305.44 | 1835.69 | 2603.18 | 1950.49 | 2386.11 | 1827.95 | 2314.28 | 2271.86 | 907.76 | 1747.90 | 2540.60 | 3238.27 | 1601.08 | 2556.12 | 3156.37 | 1348.18 | 2823.35 | 1794.38 | 2288.15 | 2924.42 | 2194.10 | 2117.48 | 1601.67 | 1343.41 | 1663.24 | 1867.41 | 1310.81 | 2696.79 | 2346.66 | 1700.47 | 2181.97 | 2446.07 |
| Geom Series df | 1.00 | 1.00 | 1.00 | 1.00 | 1.00 | 1.00 | 1.00 | 1.00 | 1.00 | 1.00 | 1.00 | 1.00 | 1.00 | 1.00 | 1.00 | 1.00 | 1.00 | 1.00 | 1.00 | 1.00 | 1.00 | 1.00 | 1.00 | 1.00 | 1.00 | 1.00 | 1.00 | 1.00 | 1.00 | 1.00 | 1.00 | 1.00 | 1.00 | 1.00 | 1.00 | 1.00 | 1.00 | 1.00 | 1.00 | 1.00 | 1.00 | 1.00 | 1.00 | 1.00 | 1.00 | 1.00 | 1.00 | 1.00 | 1.00 | 1.00 | 1.00 | 1.00 | 1.00 | 1.00 | 1.00 | 1.00 | 1.00 | 1.00 | 1.00 | 1.00 |
| Geom Series AIC weight | 0.00 | 0.00 | 0.00 | 0.00 | 0.00 | 0.00 | 0.00 | 0.00 | 0.00 | 0.00 | 0.00 | 0.00 | 0.00 | 0.00 | 0.00 | 0.00 | 0.00 | 0.00 | 0.00 | 0.00 | 0.00 | 0.00 | 0.00 | 0.00 | 0.00 | 0.00 | 0.00 | 0.00 | 0.00 | 0.00 | 0.00 | 0.00 | 0.00 | 0.00 | 0.00 | 0.00 | 0.00 | 0.00 | 0.00 | 0.00 | 0.00 | 0.00 | 0.00 | 0.00 | 0.00 | 0.00 | 0.00 | 0.00 | 0.00 | 0.00 | 0.00 | 0.00 | 0.00 | 0.00 | 0.00 | 0.00 | 0.00 | 0.00 | 0.00 | 0.00 |
| Log Series AIC | 4634.24 | 4663.87 | 4253.17 | 4710.43 | 4407.95 | 3878.48 | 5721.90 | 2574.91 | 4554.41 | 4969.71 | 5905.59 | 3622.84 | 4444.94 | 5601.67 | 5792.93 | 5628.07 | 4233.31 | 4758.47 | 5992.93 | 5499.14 | 3762.81 | 2846.36 | 4101.85 | 3805.55 | 3027.37 | 3494.26 | 3484.72 | 3553.07 | 4191.77 | 3620.60 | 4701.70 | 3384.93 | 4250.79 | 3507.95 | 4397.39 | 4281.49 | 2205.34 | 3282.82 | 5189.49 | 5729.85 | 3199.01 | 4927.82 | 5859.63 | 2839.12 | 4724.29 | 4253.15 | 4304.18 | 5287.20 | 3969.19 | 4833.33 | 2915.53 | 2649.35 | 3104.78 | 3525.21 | 2969.76 | 5194.12 | 4577.12 | 3200.27 | 3794.36 | 3988.50 |
| Log Series dAIC | 419.91 | 319.86 | 270.52 | 261.26 | 242.83 | 208.78 | 298.43 | 993.59 | 270.09 | 293.40 | 366.72 | 393.33 | 357.11 | 380.90 | 336.64 | 343.22 | 272.57 | 434.43 | 345.90 | 307.27 | 410.52 | 340.20 | 496.29 | 417.30 | 302.61 | 376.16 | 408.40 | 387.95 | 455.06 | 373.10 | 487.35 | 416.08 | 492.84 | 376.97 | 447.67 | 439.61 | 247.54 | 335.94 | 441.64 | 510.74 | 379.06 | 500.06 | 525.45 | 318.39 | 526.93 | 352.44 | 450.36 | 517.26 | 454.65 | 401.40 | 389.11 | 346.20 | 365.52 | 407.25 | 263.31 | 494.96 | 413.28 | 377.46 | 424.74 | 512.00 |
| Log Series df | 1.00 | 1.00 | 1.00 | 1.00 | 1.00 | 1.00 | 1.00 | 1.00 | 1.00 | 1.00 | 1.00 | 1.00 | 1.00 | 1.00 | 1.00 | 1.00 | 1.00 | 1.00 | 1.00 | 1.00 | 1.00 | 1.00 | 1.00 | 1.00 | 1.00 | 1.00 | 1.00 | 1.00 | 1.00 | 1.00 | 1.00 | 1.00 | 1.00 | 1.00 | 1.00 | 1.00 | 1.00 | 1.00 | 1.00 | 1.00 | 1.00 | 1.00 | 1.00 | 1.00 | 1.00 | 1.00 | 1.00 | 1.00 | 1.00 | 1.00 | 1.00 | 1.00 | 1.00 | 1.00 | 1.00 | 1.00 | 1.00 | 1.00 | 1.00 | 1.00 |
| Log Series AIC weight | 0.00 | 0.00 | 0.00 | 0.00 | 0.00 | 0.00 | 0.00 | 0.00 | 0.00 | 0.00 | 0.00 | 0.00 | 0.00 | 0.00 | 0.00 | 0.00 | 0.00 | 0.00 | 0.00 | 0.00 | 0.00 | 0.00 | 0.00 | 0.00 | 0.00 | 0.00 | 0.00 | 0.00 | 0.00 | 0.00 | 0.00 | 0.00 | 0.00 | 0.00 | 0.00 | 0.00 | 0.00 | 0.00 | 0.00 | 0.00 | 0.00 | 0.00 | 0.00 | 0.00 | 0.00 | 0.00 | 0.00 | 0.00 | 0.00 | 0.00 | 0.00 | 0.00 | 0.00 | 0.00 | 0.00 | 0.00 | 0.00 | 0.00 | 0.00 | 0.00 |
| MSZM AIC | 4637.35 | 4666.55 | 4255.73 | 4712.57 | 4409.53 | 3880.85 | 5724.20 | 2632.04 | 4556.98 | 4972.58 | 5907.76 | 3633.96 | 4448.57 | 5606.04 | 5796.19 | 5630.42 | 4235.36 | 4760.22 | 5998.58 | 5501.13 | 3766.59 | 2851.11 | 4107.00 | 3811.01 | 3030.85 | 3498.76 | 3488.36 | 3558.10 | 4196.48 | 3627.08 | 4706.30 | 3388.39 | 4255.29 | 3511.20 | 4402.22 | 4286.84 | 2208.45 | 3283.98 | 5195.66 | 5733.96 | 3206.43 | 4933.52 | 5865.01 | 2843.72 | 4729.90 | 4257.44 | 4309.12 | 5291.95 | 3974.29 | 4837.97 | 2921.70 | 2658.36 | 3114.33 | 3536.48 | 2977.33 | 5200.18 | 4582.97 | 3205.83 | 3805.58 | 3998.15 |
| MSZM dAIC | 423.02 | 322.54 | 273.09 | 263.41 | 244.41 | 211.15 | 300.73 | 1050.73 | 272.67 | 296.28 | 368.89 | 404.46 | 360.74 | 385.27 | 339.91 | 345.57 | 274.62 | 436.18 | 351.55 | 309.26 | 414.30 | 344.95 | 501.44 | 422.76 | 306.09 | 380.67 | 412.04 | 392.98 | 459.77 | 379.59 | 491.96 | 419.54 | 497.35 | 380.21 | 452.50 | 444.96 | 250.65 | 337.11 | 447.81 | 514.85 | 386.47 | 505.77 | 530.83 | 323.00 | 532.55 | 356.72 | 455.30 | 522.02 | 459.75 | 406.04 | 395.28 | 355.21 | 375.06 | 418.53 | 270.88 | 501.02 | 419.14 | 383.02 | 435.96 | 521.65 |
| MSZM df | 1.00 | 1.00 | 1.00 | 1.00 | 1.00 | 1.00 | 1.00 | 1.00 | 1.00 | 1.00 | 1.00 | 1.00 | 1.00 | 1.00 | 1.00 | 1.00 | 1.00 | 1.00 | 1.00 | 1.00 | 1.00 | 1.00 | 1.00 | 1.00 | 1.00 | 1.00 | 1.00 | 1.00 | 1.00 | 1.00 | 1.00 | 1.00 | 1.00 | 1.00 | 1.00 | 1.00 | 1.00 | 1.00 | 1.00 | 1.00 | 1.00 | 1.00 | 1.00 | 1.00 | 1.00 | 1.00 | 1.00 | 1.00 | 1.00 | 1.00 | 1.00 | 1.00 | 1.00 | 1.00 | 1.00 | 1.00 | 1.00 | 1.00 | 1.00 | 1.00 |
| MZSM AIC weight | 0.00 | 0.00 | 0.00 | 0.00 | 0.00 | 0.00 | 0.00 | 0.00 | 0.00 | 0.00 | 0.00 | 0.00 | 0.00 | 0.00 | 0.00 | 0.00 | 0.00 | 0.00 | 0.00 | 0.00 | 0.00 | 0.00 | 0.00 | 0.00 | 0.00 | 0.00 | 0.00 | 0.00 | 0.00 | 0.00 | 0.00 | 0.00 | 0.00 | 0.00 | 0.00 | 0.00 | 0.00 | 0.00 | 0.00 | 0.00 | 0.00 | 0.00 | 0.00 | 0.00 | 0.00 | 0.00 | 0.00 | 0.00 | 0.00 | 0.00 | 0.00 | 0.00 | 0.00 | 0.00 | 0.00 | 0.00 | 0.00 | 0.00 | 0.00 | 0.00 |
| Pareto AIC | 4214.33 | 4344.01 | 3982.64 | 4449.17 | 4165.11 | 3669.70 | 5423.47 | 1581.32 | 4284.31 | 4676.30 | 5538.87 | 3229.50 | 4087.83 | 5220.77 | 5456.29 | 5284.85 | 3960.74 | 4324.04 | 5647.04 | 5191.87 | 3352.29 | 2506.16 | 3605.56 | 3388.25 | 2724.77 | 3118.09 | 3076.31 | 3165.12 | 3736.71 | 3247.50 | 4214.35 | 2968.85 | 3757.95 | 3130.99 | 3949.71 | 3841.88 | 1957.80 | 2946.87 | 4747.84 | 5219.11 | 2819.95 | 4427.75 | 5334.18 | 2520.72 | 4197.36 | 3900.71 | 3853.82 | 4769.94 | 3514.54 | 4431.93 | 2526.42 | 2303.15 | 2739.26 | 3117.96 | 2706.45 | 4699.16 | 4163.83 | 2822.81 | 3369.62 | 3476.50 |
| Pareto dAIC | 0.00 | 0.00 | 0.00 | 0.00 | 0.00 | 0.00 | 0.00 | 0.00 | 0.00 | 0.00 | 0.00 | 0.00 | 0.00 | 0.00 | 0.00 | 0.00 | 0.00 | 0.00 | 0.00 | 0.00 | 0.00 | 0.00 | 0.00 | 0.00 | 0.00 | 0.00 | 0.00 | 0.00 | 0.00 | 0.00 | 0.00 | 0.00 | 0.00 | 0.00 | 0.00 | 0.00 | 0.00 | 0.00 | 0.00 | 0.00 | 0.00 | 0.00 | 0.00 | 0.00 | 0.00 | 0.00 | 0.00 | 0.00 | 0.00 | 0.00 | 0.00 | 0.00 | 0.00 | 0.00 | 0.00 | 0.00 | 0.00 | 0.00 | 0.00 | 0.00 |
| Pareto df | 2.00 | 2.00 | 2.00 | 2.00 | 2.00 | 2.00 | 2.00 | 2.00 | 2.00 | 2.00 | 2.00 | 2.00 | 2.00 | 2.00 | 2.00 | 2.00 | 2.00 | 2.00 | 2.00 | 2.00 | 2.00 | 2.00 | 2.00 | 2.00 | 2.00 | 2.00 | 2.00 | 2.00 | 2.00 | 2.00 | 2.00 | 2.00 | 2.00 | 2.00 | 2.00 | 2.00 | 2.00 | 2.00 | 2.00 | 2.00 | 2.00 | 2.00 | 2.00 | 2.00 | 2.00 | 2.00 | 2.00 | 2.00 | 2.00 | 2.00 | 2.00 | 2.00 | 2.00 | 2.00 | 2.00 | 2.00 | 2.00 | 2.00 | 2.00 | 2.00 |
| Pareto AIC weight | 1.00 | 1.00 | 1.00 | 1.00 | 1.00 | 1.00 | 1.00 | 1.00 | 1.00 | 1.00 | 1.00 | 1.00 | 1.00 | 1.00 | 1.00 | 1.00 | 1.00 | 1.00 | 1.00 | 1.00 | 1.00 | 1.00 | 1.00 | 1.00 | 1.00 | 1.00 | 1.00 | 1.00 | 1.00 | 1.00 | 1.00 | 1.00 | 1.00 | 1.00 | 1.00 | 1.00 | 1.00 | 1.00 | 1.00 | 1.00 | 1.00 | 1.00 | 1.00 | 1.00 | 1.00 | 1.00 | 1.00 | 1.00 | 1.00 | 1.00 | 1.00 | 1.00 | 1.00 | 1.00 | 1.00 | 1.00 | 1.00 | 1.00 | 1.00 | 1.00 |
| Pois-Log AIC | 4492.93 | 4575.61 | 4185.02 | 4662.76 | 4358.73 | 3833.93 | 5652.48 | 1973.72 | 4487.63 | 4898.79 | 5798.30 | 3461.82 | 4326.97 | 5493.22 | 5697.91 | 5534.66 | 4164.74 | 4611.01 | 5875.12 | 5424.86 | 3611.23 | 2717.72 | 3897.28 | 3639.97 | 2921.44 | 3353.77 | 3317.92 | 3409.50 | 4011.47 | 3474.94 | 4505.64 | 3222.43 | 4054.46 | 3368.31 | 4217.25 | 4114.36 | 2132.67 | 3186.66 | 5016.09 | 5515.47 | 3043.09 | 4720.68 | 5625.43 | 2727.45 | 4482.84 | 4132.57 | 4124.89 | 5065.69 | 3787.53 | 4692.19 | 2758.69 | 2519.63 | 2953.64 | 3354.25 | 2866.34 | 4991.27 | 4399.16 | 3057.59 | 3609.00 | 3752.68 |
| Pois-Log dAIC | 278.60 | 231.60 | 202.38 | 213.60 | 193.62 | 164.23 | 229.01 | 392.40 | 203.32 | 222.48 | 259.43 | 232.31 | 239.13 | 272.45 | 241.62 | 249.81 | 204.00 | 286.97 | 228.08 | 232.98 | 258.94 | 211.56 | 291.72 | 251.72 | 196.67 | 235.68 | 241.60 | 244.38 | 274.76 | 227.45 | 291.29 | 253.58 | 296.51 | 237.32 | 267.53 | 272.48 | 174.88 | 239.78 | 268.25 | 296.36 | 223.13 | 292.93 | 291.25 | 206.72 | 285.49 | 231.86 | 271.06 | 295.75 | 272.99 | 260.25 | 232.27 | 216.49 | 214.38 | 236.29 | 159.89 | 292.11 | 235.33 | 234.78 | 239.38 | 276.18 |
| Pois-Log df | 2.00 | 2.00 | 2.00 | 2.00 | 2.00 | 2.00 | 2.00 | 2.00 | 2.00 | 2.00 | 2.00 | 2.00 | 2.00 | 2.00 | 2.00 | 2.00 | 2.00 | 2.00 | 2.00 | 2.00 | 2.00 | 2.00 | 2.00 | 2.00 | 2.00 | 2.00 | 2.00 | 2.00 | 2.00 | 2.00 | 2.00 | 2.00 | 2.00 | 2.00 | 2.00 | 2.00 | 2.00 | 2.00 | 2.00 | 2.00 | 2.00 | 2.00 | 2.00 | 2.00 | 2.00 | 2.00 | 2.00 | 2.00 | 2.00 | 2.00 | 2.00 | 2.00 | 2.00 | 2.00 | 2.00 | 2.00 | 2.00 | 2.00 | 2.00 | 2.00 |
| Pois-log AIC weight | 0.00 | 0.00 | 0.00 | 0.00 | 0.00 | 0.00 | 0.00 | 0.00 | 0.00 | 0.00 | 0.00 | 0.00 | 0.00 | 0.00 | 0.00 | 0.00 | 0.00 | 0.00 | 0.00 | 0.00 | 0.00 | 0.00 | 0.00 | 0.00 | 0.00 | 0.00 | 0.00 | 0.00 | 0.00 | 0.00 | 0.00 | 0.00 | 0.00 | 0.00 | 0.00 | 0.00 | 0.00 | 0.00 | 0.00 | 0.00 | 0.00 | 0.00 | 0.00 | 0.00 | 0.00 | 0.00 | 0.00 | 0.00 | 0.00 | 0.00 | 0.00 | 0.00 | 0.00 | 0.00 | 0.00 | 0.00 | 0.00 | 0.00 | 0.00 | 0.00 |
| Lognormal AIC | 4803.21 | 4826.49 | 4388.23 | 4881.71 | 4549.00 | 3978.95 | 5910.56 | 2287.34 | 4690.68 | 5133.72 | 6083.73 | 3682.05 | 4589.59 | 5794.12 | 5950.87 | 5807.22 | 4374.16 | 4949.45 | 6124.65 | 5687.43 | 3845.88 | 2873.02 | 4188.63 | 3886.46 | 3099.59 | 3558.92 | 3532.80 | 3617.59 | 4273.35 | 3665.64 | 4788.35 | 3469.84 | 4334.75 | 3586.94 | 4459.21 | 4374.74 | 2245.47 | 3459.92 | 5259.72 | 5827.57 | 3195.60 | 4968.53 | 5897.81 | 2886.03 | 4745.14 | 4311.77 | 4370.60 | 5349.61 | 4038.29 | 4903.14 | 2940.05 | 2674.68 | 3123.59 | 3533.99 | 2980.84 | 5254.72 | 4602.66 | 3263.14 | 3822.83 | 3990.55 |
| Lognormal dAIC | 588.88 | 482.49 | 405.59 | 432.55 | 383.88 | 309.25 | 487.10 | 706.02 | 406.37 | 457.42 | 544.86 | 452.55 | 501.76 | 573.35 | 494.58 | 522.36 | 413.42 | 625.41 | 477.61 | 495.56 | 493.60 | 366.85 | 583.07 | 498.21 | 374.83 | 440.83 | 456.49 | 452.47 | 536.64 | 418.14 | 574.00 | 500.99 | 576.81 | 455.95 | 509.49 | 532.86 | 287.67 | 513.04 | 511.88 | 608.46 | 375.65 | 540.78 | 563.63 | 365.31 | 547.78 | 411.06 | 516.77 | 579.68 | 523.75 | 471.20 | 413.62 | 371.53 | 384.32 | 416.03 | 274.39 | 555.56 | 438.83 | 440.33 | 453.21 | 514.04 |
| Lognormal df | 2.00 | 2.00 | 2.00 | 2.00 | 2.00 | 2.00 | 2.00 | 2.00 | 2.00 | 2.00 | 2.00 | 2.00 | 2.00 | 2.00 | 2.00 | 2.00 | 2.00 | 2.00 | 2.00 | 2.00 | 2.00 | 2.00 | 2.00 | 2.00 | 2.00 | 2.00 | 2.00 | 2.00 | 2.00 | 2.00 | 2.00 | 2.00 | 2.00 | 2.00 | 2.00 | 2.00 | 2.00 | 2.00 | 2.00 | 2.00 | 2.00 | 2.00 | 2.00 | 2.00 | 2.00 | 2.00 | 2.00 | 2.00 | 2.00 | 2.00 | 2.00 | 2.00 | 2.00 | 2.00 | 2.00 | 2.00 | 2.00 | 2.00 | 2.00 | 2.00 |
| Lognormal AIC weight | 0.00 | 0.00 | 0.00 | 0.00 | 0.00 | 0.00 | 0.00 | 0.00 | 0.00 | 0.00 | 0.00 | 0.00 | 0.00 | 0.00 | 0.00 | 0.00 | 0.00 | 0.00 | 0.00 | 0.00 | 0.00 | 0.00 | 0.00 | 0.00 | 0.00 | 0.00 | 0.00 | 0.00 | 0.00 | 0.00 | 0.00 | 0.00 | 0.00 | 0.00 | 0.00 | 0.00 | 0.00 | 0.00 | 0.00 | 0.00 | 0.00 | 0.00 | 0.00 | 0.00 | 0.00 | 0.00 | 0.00 | 0.00 | 0.00 | 0.00 | 0.00 | 0.00 | 0.00 | 0.00 | 0.00 | 0.00 | 0.00 | 0.00 | 0.00 | 0.00 |
| Power AIC | 4483.99 | 4588.28 | 4210.86 | 4691.77 | 4394.33 | 3878.91 | 5682.80 | 1953.26 | 4520.20 | 4925.63 | 5825.18 | 3471.89 | 4330.16 | 5507.35 | 5738.33 | 5559.60 | 4186.57 | 4585.52 | 5916.98 | 5449.69 | 3624.22 | 2739.21 | 3895.61 | 3645.61 | 2933.84 | 3370.36 | 3330.94 | 3427.59 | 4022.71 | 3499.00 | 4519.11 | 3220.48 | 4062.49 | 3379.00 | 4242.33 | 4127.10 | 2154.57 | 3163.96 | 5058.83 | 5534.73 | 3077.90 | 4758.87 | 5673.10 | 2746.81 | 4505.46 | 4182.54 | 4145.43 | 5094.30 | 3799.09 | 4743.19 | 2772.51 | 2536.81 | 2974.13 | 3382.20 | 2907.51 | 5027.58 | 4441.88 | 3067.08 | 3626.33 | 3770.73 |
| Power dAIC | 269.66 | 244.27 | 228.22 | 242.60 | 229.22 | 209.21 | 259.33 | 371.94 | 235.89 | 249.33 | 286.30 | 242.39 | 242.33 | 286.58 | 282.04 | 274.75 | 225.83 | 261.48 | 269.95 | 257.82 | 271.94 | 233.04 | 290.05 | 257.37 | 209.08 | 252.27 | 254.63 | 262.47 | 286.00 | 251.50 | 304.77 | 251.63 | 304.54 | 248.01 | 292.62 | 285.22 | 196.77 | 217.08 | 310.99 | 315.62 | 257.94 | 331.12 | 338.92 | 226.08 | 308.10 | 281.83 | 291.61 | 324.36 | 284.55 | 311.25 | 246.09 | 233.67 | 234.87 | 264.24 | 201.06 | 328.42 | 278.05 | 244.27 | 256.71 | 294.23 |
| Power df | 1.00 | 1.00 | 1.00 | 1.00 | 1.00 | 1.00 | 1.00 | 1.00 | 1.00 | 1.00 | 1.00 | 1.00 | 1.00 | 1.00 | 1.00 | 1.00 | 1.00 | 1.00 | 1.00 | 1.00 | 1.00 | 1.00 | 1.00 | 1.00 | 1.00 | 1.00 | 1.00 | 1.00 | 1.00 | 1.00 | 1.00 | 1.00 | 1.00 | 1.00 | 1.00 | 1.00 | 1.00 | 1.00 | 1.00 | 1.00 | 1.00 | 1.00 | 1.00 | 1.00 | 1.00 | 1.00 | 1.00 | 1.00 | 1.00 | 1.00 | 1.00 | 1.00 | 1.00 | 1.00 | 1.00 | 1.00 | 1.00 | 1.00 | 1.00 | 1.00 |
| Power AIC weight | 0.00 | 0.00 | 0.00 | 0.00 | 0.00 | 0.00 | 0.00 | 0.00 | 0.00 | 0.00 | 0.00 | 0.00 | 0.00 | 0.00 | 0.00 | 0.00 | 0.00 | 0.00 | 0.00 | 0.00 | 0.00 | 0.00 | 0.00 | 0.00 | 0.00 | 0.00 | 0.00 | 0.00 | 0.00 | 0.00 | 0.00 | 0.00 | 0.00 | 0.00 | 0.00 | 0.00 | 0.00 | 0.00 | 0.00 | 0.00 | 0.00 | 0.00 | 0.00 | 0.00 | 0.00 | 0.00 | 0.00 | 0.00 | 0.00 | 0.00 | 0.00 | 0.00 | 0.00 | 0.00 | 0.00 | 0.00 | 0.00 | 0.00 | 0.00 | 0.00 |
| Weibull AIC | 5004.70 | 4998.79 | 4544.71 | 5033.27 | 4703.89 | 4121.85 | 6087.74 | 2692.09 | 4852.61 | 5303.12 | 6296.29 | 3875.50 | 4768.20 | 5995.24 | 6154.90 | 6000.64 | 4531.67 | 5150.84 | 6331.87 | 5869.09 | 4061.31 | 3065.10 | 4427.32 | 4093.24 | 3262.70 | 3760.90 | 3753.47 | 3826.34 | 4507.52 | 3871.05 | 5041.07 | 3670.29 | 4584.54 | 3784.26 | 4710.27 | 4599.30 | 2392.42 | 3603.79 | 5519.28 | 6104.98 | 3420.57 | 5254.85 | 6210.17 | 3063.41 | 5028.80 | 4534.29 | 4612.90 | 5637.44 | 4271.09 | 5151.99 | 3146.26 | 2859.91 | 3321.27 | 3755.66 | 3149.94 | 5536.37 | 4850.45 | 3454.78 | 4039.01 | 4257.54 |
| Weibull dAIC | 790.37 | 654.78 | 562.07 | 584.11 | 538.77 | 452.15 | 664.27 | 1110.78 | 568.30 | 626.82 | 757.42 | 646.00 | 680.37 | 774.47 | 698.61 | 715.79 | 570.93 | 826.80 | 684.83 | 677.22 | 709.03 | 558.94 | 821.76 | 705.00 | 537.93 | 642.80 | 677.16 | 661.22 | 770.81 | 623.55 | 826.72 | 701.44 | 826.59 | 653.27 | 760.55 | 757.42 | 434.62 | 656.92 | 771.44 | 885.88 | 600.61 | 827.09 | 875.99 | 542.69 | 831.45 | 633.58 | 759.08 | 867.51 | 756.55 | 720.06 | 619.84 | 556.77 | 582.01 | 637.70 | 443.49 | 837.21 | 686.61 | 631.97 | 669.39 | 781.04 |
| Weibull df | 2.00 | 2.00 | 2.00 | 2.00 | 2.00 | 2.00 | 2.00 | 2.00 | 2.00 | 2.00 | 2.00 | 2.00 | 2.00 | 2.00 | 2.00 | 2.00 | 2.00 | 2.00 | 2.00 | 2.00 | 2.00 | 2.00 | 2.00 | 2.00 | 2.00 | 2.00 | 2.00 | 2.00 | 2.00 | 2.00 | 2.00 | 2.00 | 2.00 | 2.00 | 2.00 | 2.00 | 2.00 | 2.00 | 2.00 | 2.00 | 2.00 | 2.00 | 2.00 | 2.00 | 2.00 | 2.00 | 2.00 | 2.00 | 2.00 | 2.00 | 2.00 | 2.00 | 2.00 | 2.00 | 2.00 | 2.00 | 2.00 | 2.00 | 2.00 | 2.00 |
| Weibull AIC weight | 0.00 | 0.00 | 0.00 | 0.00 | 0.00 | 0.00 | 0.00 | 0.00 | 0.00 | 0.00 | 0.00 | 0.00 | 0.00 | 0.00 | 0.00 | 0.00 | 0.00 | 0.00 | 0.00 | 0.00 | 0.00 | 0.00 | 0.00 | 0.00 | 0.00 | 0.00 | 0.00 | 0.00 | 0.00 | 0.00 | 0.00 | 0.00 | 0.00 | 0.00 | 0.00 | 0.00 | 0.00 | 0.00 | 0.00 | 0.00 | 0.00 | 0.00 | 0.00 | 0.00 | 0.00 | 0.00 | 0.00 | 0.00 | 0.00 | 0.00 | 0.00 | 0.00 | 0.00 | 0.00 | 0.00 | 0.00 | 0.00 | 0.00 | 0.00 | 0.00 |
