# Supplementary material for: Microbial micropatches within microbial hotspots
Source: PLoS One. 2018 May 22;13(5):e0197224. doi: 10.1371/journal.pone.0197224 (PMC5963804; doi:10.1371/journal.pone.0197224)
Supplement: S2 Table — (DOCX) [file pone.0197224.s008.docx]

**S2 Table.** MLE-calculated optimal Pareto (power) law truncation point, estimated best-fit exponent, and goodness of fit test information for genus abundance distributions across all samples.

| **Sample** | **Shape parameter (all data)** | **xmin** | **Truncated power exponent** | **KS Statistic** | **KS Critical Value** | **Hypothesis test logical** | **Mean exponent** |
| --- | --- | --- | --- | --- | --- | --- | --- |
| H1.1 | 0.498 | 259 | -2.02 | 0.066 | 0.111 | 0 |  |
| H1.2 | 0.461 | 276 | -2.03 | 0.073 | 0.115 | 0 |  |
| H1.3 | 0.466 | 94 | -1.92 | 0.045 | 0.090 | 0 |  |
| H1.4 | 0.452 | 96 | -1.94 | 0.054 | 0.083 | 0 |  |
| H1.5 | 0.455 | 94 | -1.89 | 0.053 | 0.088 | 0 |  |
| H1.6 | 0.465 | 66 | -1.85 | 0.058 | 0.093 | 0 |  |
| H1.7 | 0.418 | 293 | -1.99 | 0.062 | 0.101 | 0 |  |
| H1.8 | 1.071 | 1 | -1.72 | 0.029 | 0.040 | 0 |  |
| H1.9 | 0.455 | 143 | -2 | 0.043 | 0.100 | 0 |  |
| H1.10 | 0.446 | 168 | -1.95 | 0.054 | 0.096 | 0 |  |
| H2.1 | 0.438 | 109 | -1.8 | 0.059 | 0.074 | 0 |  |
| H2.2 | 0.548 | 68 | -1.87 | 0.074 | 0.100 | 0 |  |
| H2.3 | 0.476 | 114 | -1.92 | 0.050 | 0.086 | 0 |  |
| H2.4 | 0.454 | 75 | -1.8 | 0.053 | 0.068 | 0 |  |
| H2.5 | 0.438 | 172 | -1.9 | 0.060 | 0.092 | 0 |  |
| H3.1 | 0.439 | 223 | -1.99 | 0.053 | 0.096 | 0 |  |
| H3.2 | 0.465 | 24 | -1.61 | 0.090 | 0.065 | 1 |  |
| H3.3 | 0.481 | 86 | -1.67 | 0.086 | 0.076 | 1 |  |
| H3.4 | 0.418 | 63 | -1.74 | 0.046 | 0.063 | 0 | Hotspots |
| H3.5 | 0.427 | 158 | -1.86 | 0.073 | 0.083 | 0 | -1.90 |
| C1.1 | 0.574 | 21 | -1.7 | 0.050 | 0.074 | 0 |  |
| C1.2 | 0.624 | 11 | -1.77 | 0.052 | 0.074 | 0 |  |
| C1.3 | 0.571 | 92 | -1.8 | 0.061 | 0.104 | 0 |  |
| C1.4 | 0.552 | 46 | -1.8 | 0.051 | 0.085 | 0 |  |
| C1.5 | 0.556 | 74 | -1.86 | 0.062 | 0.115 | 0 |  |
| C1.6 | 0.574 | 33 | -1.79 | 0.046 | 0.087 | 0 |  |
| C1.7 | 0.582 | 38 | -1.72 | 0.061 | 0.094 | 0 |  |
| C1.8 | 0.582 | 14 | -1.7 | 0.047 | 0.069 | 0 |  |
| C1.9 | 0.554 | 37 | -1.71 | 0.067 | 0.080 | 0 |  |
| C1.10 | 0.559 | 34 | -1.81 | 0.035 | 0.088 | 0 |  |
| C2.1 | 0.536 | 42 | -1.69 | 0.048 | 0.079 | 0 |  |
| C2.2 | 0.590 | 44 | -1.76 | 0.053 | 0.094 | 0 |  |
| C2.3 | 0.574 | 70 | -1.81 | 0.056 | 0.097 | 0 |  |
| C2.4 | 0.566 | 21 | -1.73 | 0.037 | 0.074 | 0 |  |
| C2.5 | 0.544 | 67 | -1.76 | 0.068 | 0.096 | 0 |  |
| C3.1 | 0.544 | 57 | -1.84 | 0.038 | 0.086 | 0 |  |
| C3.2 | 0.655 | 9 | -1.81 | 0.058 | 0.080 | 0 |  |
| C3.3 | 0.543 | 514 | -2.7 | 0.083 | 0.182 | 0 |  |
| C3.4 | 0.505 | 60 | -1.81 | 0.045 | 0.080 | 0 |  |
| C3.5 | 0.481 | 170 | -1.84 | 0.066 | 0.093 | 0 | Coldspots |
| B1.1 | 0.618 | 8 | -1.76 | 0.039 | 0.064 | 0 | -1.77 |
| B1.2 | 0.546 | 37 | -1.74 | 0.058 | 0.078 | 0 |  |
| B1.3 | 0.495 | 212 | -1.85 | 0.056 | 0.114 | 0 |  |
| B1.4 | 0.611 | 43 | -1.9 | 0.061 | 0.112 | 0 |  |
| B1.5 | 0.540 | 16 | -1.64 | 0.034 | 0.062 | 0 |  |
| B1.6 | 0.536 | 15 | -1.75 | 0.048 | 0.062 | 0 |  |
| B1.7 | 0.551 | 60 | -1.81 | 0.054 | 0.093 | 0 |  |
| B1.8 | 0.516 | 41 | -1.71 | 0.045 | 0.071 | 0 |  |
| B1.9 | 0.574 | 113 | -1.9 | 0.057 | 0.120 | 0 |  |
| B1.10 | 0.526 | 15 | -1.68 | 0.055 | 0.059 | 0 |  |
| B2.1 | 0.641 | 22 | -1.83 | 0.065 | 0.093 | 0 |  |
| B2.2 | 0.658 | 15 | -1.82 | 0.058 | 0.087 | 0 |  |
| B2.3 | 0.594 | 10 | -1.73 | 0.039 | 0.067 | 0 |  |
| B2.4 | 0.590 | 22 | -1.82 | 0.053 | 0.081 | 0 |  |
| B2.5 | 0.546 | 21 | -1.85 | 0.049 | 0.084 | 0 |  |
| B3.1 | 0.525 | 86 | -1.86 | 0.055 | 0.090 | 0 |  |
| B3.2 | 0.511 | 14 | -1.65 | 0.046 | 0.060 | 0 |  |
| B3.3 | 0.597 | 17 | -1.73 | 0.043 | 0.077 | 0 |  |
| B3.4 | 0.553 | 46 | -1.8 | 0.050 | 0.093 | 0 | Background |
| B3.5 | 0.589 | 16 | -1.69 | 0.030 | 0.070 | 0 | -1.78 |
