# Supplementary material for: Microbial micropatches within microbial hotspots
Source: PLoS One. 2018 May 22;13(5):e0197224. doi: 10.1371/journal.pone.0197224 (PMC5963804; doi:10.1371/journal.pone.0197224)
Supplement: S3 Table — (DOCX) [file pone.0197224.s009.docx]

**S3 Table.** Total prokaryotic abundances determined via flow cytometry.

| **Sample** | **Abundance**  x 10^7^ cells ml^-1^ (95%CI, n) |
| --- | --- |
| **H1** | 4.6 (0.7 x 10^7^) |
| **H2** | 4.5 (1.9 x 10^7^) |
| **H3** | 4.3 (0.8 x 10^7^) |
| **C1** | 0.92 (1.7 x 10^6^) |
| **C2 and C3** | 0.94 (3.3 x 10^6^) |
| **B1, B2 and B3** | 2.7 (0.9 x 10^7^) |
