# Supplementary material for: Microbial micropatches within microbial hotspots
Source: PLoS One. 2018 May 22;13(5):e0197224. doi: 10.1371/journal.pone.0197224 (PMC5963804; doi:10.1371/journal.pone.0197224)
Supplement: S4 Table — (DOCX) [file pone.0197224.s010.docx]

**S4 Table.** SIMPER similarity comparisons between subsamples with no removal and removal of genera with heightened relative abundance.

| **Samples** | **SIMPER Similarity** | |
| --- | --- | --- |
|  | **No removal** | **Genera with heightened abundance removed** |
| C2 | 70.3 | 70.7 |
| C3 | 66.0 | 66.3 |
| C1 | 71.6 | 71.8 |
| H2 | 57.5 | 58.2 |
| H3 | 61.2 | 61.6 |
| H1 | 53.3 | 53.9 |
| B2 | 74.6 | 75.2 |
| B3 | 74.1 | 74.9 |
| B1 | 76.2 | 76.6 |
