# Supplementary material for: Microbial micropatches within microbial hotspots
Source: PLoS One. 2018 May 22;13(5):e0197224. doi: 10.1371/journal.pone.0197224 (PMC5963804; doi:10.1371/journal.pone.0197224)
Supplement: S5 Table — (DOCX) [file pone.0197224.s011.docx]

**S5 Table.** SIMPER similarity comparisons between samples with no removal and removal of genera with heightened relative abundance.

| **Samples** | **SIMPER Dissimilarity** | |
| --- | --- | --- |
|  | **No removal** | **Genera with heightened abundance removed** |
| C2 & C3 | 31.7 | 31.4 |
| C2 & C1 | 29.2 | 28.8 |
| C3 & C1 | 31.4 | 31.2 |
| C2 & H2 | 47.2 | 47.1 |
| C3 & H2 | 48.5 | 48.3 |
| C1 & H2 | 46.1 | 45.8 |
| C2 & H3 | 45.5 | 44.8 |
| C3 & H3 | 47.3 | 46.5 |
| C1 & H3 | 45.2 | 44.3 |
| H2 & H3 | 43.4 | 41.8 |
| C2 & H1 | 48.4 | 48.2 |
| C3 & H1 | 49.4 | 49.1 |
| C1& H1 | 47.3 | 46.9 |
| H1 & H2 | 46.5 | 45.9 |
| H3 & H1 | 42.9 | 42.7 |
| C2 & B2 | 40.6 | 40.2 |
| C3 & B2 | 42.0 | 41.8 |
| C1 & B2 | 39.1 | 38.9 |
| H2 & B2 | 49.8 | 49.4 |
| H3 & B2 | 50.0 | 49.0 |
| H1 & B2 | 52.3 | 51.8 |
| C2 & B3 | 40.5 | 40.4 |
| C3 & B3 | 41.8 | 41.6 |
| C1 & B3 | 39.2 | 39.0 |
| H2 & B3 | 50.1 | 49.9 |
| H3 & B3 | 50.9 | 49.8 |
| H1 & B3 | 53.1 | 52.7 |
| B2 & B3 | 26.6 | 25.9 |
| C2 & B1 | 39.1 | 39.0 |
| C3 & B1 | 40.5 | 40.3 |
| C1 & B1 | 38.3 | 38.0 |
| H2 & B1 | 49.1 | 48.9 |
| H3 & B1 | 49.9 | 48.8 |
| H1 & B1 | 51.5 | 51.0 |
| B2 & B1 | 27.2 | 26.6 |
| B3 & B1 | 25.5 | 25.0 |
